# Non-equilibrium crystallization and spatial fingerprints in flash IR-annealed halide perovskite films

**DOI:** 10.1039/d6mh00916f

**Published:** 2026-07-17

**Authors:** Ornella Vaccarelli, Tiziano Agostino Caldara, Christophe Gisler, Jean Hennebert, Sandy Sánchez Alonso

**Affiliations:** a Institute of AI and Complex Systems (iCoSys), School of Engineering and Architecture of Fribourg (HEIA-FR), University of Applied Sciences and Arts Western Switzerland (HES-SO) 1700 Fribourg Switzerland; b Laboratory of Photonics and Interfaces, Institute of Chemistry and Chemical Engineering, École Polytechnique Fédérale de Lausanne 1015 Lausanne Switzerland; c Institute of Smart and Secured Systems (iSIS), School of Engineering and Architecture of Fribourg (HEIA-FR), University of Applied Sciences and Arts Western Switzerland (HES-SO) 1700 Fribourg Switzerland sandy.sanchez@hefr.ch

## Abstract

Metal–halide perovskite solar cells combine high power-conversion efficiencies with solution processability, yet scalable fabrication remains limited by incomplete control over crystallization pathways and the resulting film heterogeneity. Under flash infrared annealing (FIRA), millisecond photonic pulses drive strongly non-equilibrium nucleation and growth, producing spherulitic microstructures whose final geometry stores measurable comparative signatures of the underlying crystallization pathway. Here, we establish a segmentation-based framework that converts bright-field microscopy of FIRA-processed films into quantitative comparative descriptors of grain morphology, video-anchored effective kinetics, and spatial microstructural fingerprints, providing a practical route to analyze crystallization under manufacturing-relevant rapid-processing conditions. Using time-resolved crystallization videos of pristine FAPI and FAPI–TEMPO together with a larger static microscopy dataset of roughly 3000 images from about 100 processed films, we quantify how additive chemistry reorganizes crystallization across both dynamic and end-state image populations. The workflow combines semi-supervised instance segmentation and mask-quality classification with grain-level morphology extraction and video-anchored kinetic reconstruction, with the video data providing the kinetic anchor and the static dataset providing the principal statistical support. From a curated library of more than 420 000 validated spherulites (180 545 for FAPI and 241 619 for FAPI–TEMPO), we derive effective growth-rate distributions, transformed-fraction curves, empirical kinetic descriptors, and spatial signatures based on texture entropy, defect loading, shape regularity, radial profiles, and crowding metrics. We find that TEMPO delays and narrows the dominant crystallization burst, reduces grain-size dispersion (median area reduced by 34%, *Δ* = 357 µm^2^, Cliff's *δ* = 0.62), reduces optically defect-like outer-front heterogeneity, and contracts the accessible kinetic landscape while preserving the overall spherulitic growth motif. Sample-level nonparametric statistics further show that area, perimeter, equivalent radius, and the effective growth rate are all larger in pristine FAPI, whereas the whole-grain texture entropy (*h*_m_) is comparable between the two compositions, indicating that the additive redistributes intragrain disorder spatially rather than changing its total amount. These results are consistent with additive-mediated narrowing of the accessible crystallization pathway under rapid annealing. More broadly, the workflow shows that bright-field imaging can serve as a scalable probe of non-equilibrium crystallization in solution-processed semiconductors and provides a transferable route for linking processing, crystallization dynamics, and final microstructure in rapidly solidified thin films.

## Abstract 2

Metal–halide perovskite solar cells combine high power-conversion efficiencies with solution processability, yet scalable fabrication remains limited by incomplete control over crystallization pathways and the resulting film heterogeneity.

## Graphical abstract

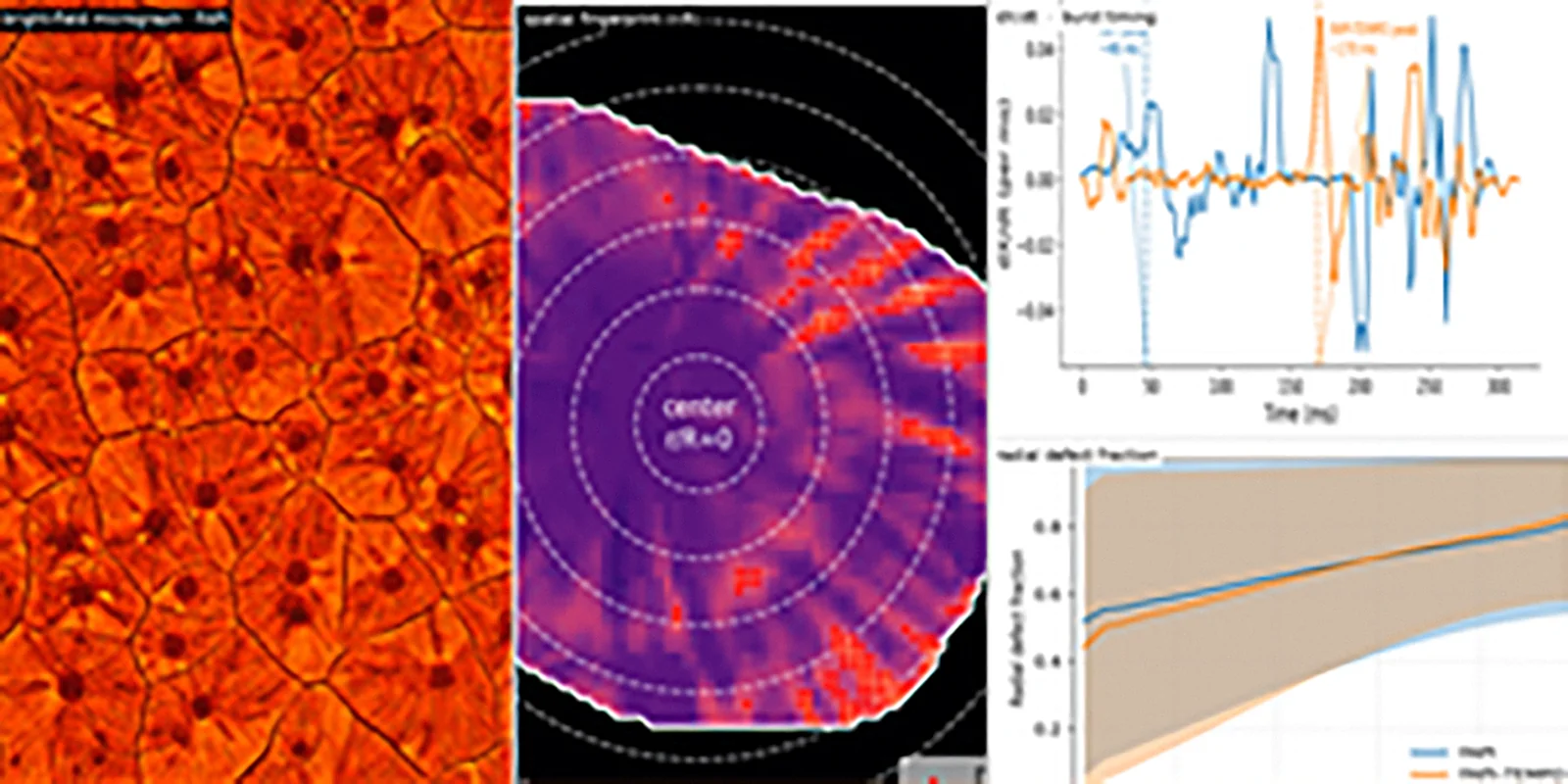

**New concepts**

Existing studies of perovskite additive engineering usually infer crystallization pathways from device performance, bulk structural probes, or static microscopy. This work introduces a different concept: the final bright-field optical microstructure can be treated as a quantitative, statistically sampled record of the non-equilibrium crystallization trajectory. We demonstrate this by converting FIRA-grown FAPI and FAPI–TEMPO films into curated grain populations and linking time-resolved videos with thousands of end-state grains through segmentation-informed morphology, effective kinetics, radial fingerprints, and crowding descriptors. The breakthrough is not a new imaging modality, but a new materials-analysis framework that makes low-cost optical microscopy report on additive-controlled rapid solidification at the grain-population level. Unlike conventional image analysis that measures only size or shape distributions, the approach maps where kinetic and defect-related signatures are stored within grains, especially at the advancing outer front. This provides materials-science insight into how a low-loading nitroxide additive reshapes, rather than simply slows, crystallization: TEMPO narrows the accessible kinetic landscape while preserving the spherulitic growth class. The concept should be transferable to other solution-processed semiconductors and rapidly solidified thin films where large image datasets exist but statistically resolved structure–processing links are difficult to obtain.

## Introduction

Solution-processed metal–halide perovskites have rapidly become a leading materials platform for next-generation optoelectronic devices, including high-efficiency solar cells, light-emitting diodes, photodetectors, and radiation detectors.^21,22,31,62,77,79^ As the field moves from record small-area efficiencies toward robust manufacturing, morphology control, scalable processing, and film homogeneity have become as important as device performance itself.^32,49,94^ This sensitivity arises because the favorable optoelectronic properties of halide perovskites are coupled to soft ionic lattices and unusual defect physics, making film formation highly responsive to processing history rather than composition alone.^13,28,55,61,86,90,95^

In practice, perovskite films are rarely formed under equilibrium conditions. Scalable deposition instead relies on highly driven crystallization pathways, including antisolvent-assisted deposition, sequential conversion, blade coating, slot-die coating, gas quenching, rapid thermal treatment, and photonic annealing.^1,16,36,44,45,57,87,92^ Under such conditions, solvent evaporation, supersaturation, intermediate-phase evolution, nucleation, growth, and grain impingement occur on overlapping timescales and couple strongly to precursor stoichiometry, solvent coordination, additive chemistry, and drying-field control.^9,17,30,35,88,89,93,97^ Recent manufacturing studies further show that solvent choice, co-deposition, vapor partitioning, synchronized multication growth, and intermediate-phase control, as well as scalable coating routes that tune crystallographic orientation and film organization, can strongly reshape crystallization windows and film homogeneity in scalable processing.^15,20,41,42,47,72,96,98,99,102,103^ The final film should therefore be viewed as a microstructural record of a strongly path-dependent solidification process rather than simply a thermodynamic endpoint.^11,38,59,60,105^

A particularly relevant example is photonic annealing on millisecond-to-subsecond timescales. In flash infrared annealing (FIRA), a short, intense thermal pulse drives ultrafast solvent removal, supersaturation, nucleation, and crystal growth.^46,64,66–69,75,83^ This regime offers clear technological advantages, including compatibility with flexible substrates, reduced thermal budgets, and high-throughput manufacturing.^52,65,66,71^ Related rapid-processing routes, including laser annealing, temperature-controlled vacuum quenching, and pressure-assisted annealing, further highlight the broader relevance of transient thermal pathways for perovskite manufacturing.^5,12,29^ At the same time, the strong thermal transient drives crystallization far from equilibrium, so the resulting spherulitic grain structure is expected to retain a complex signature of precursor chemistry, additive action, and annealing history.^11,38^ Direct per-grain quantification of how additive chemistry reorganizes the FIRA crystallization pathway, however, has not previously been reported.

Chemical additives are central to controlling this pathway. Lewis bases, alkali salts, ionic species, molecular passivators, and supramolecular ligands have all been used to regulate precursor interactions, stabilize intermediates, alter nucleation barriers, tune growth, and suppress defect formation.^40,43,48,56,58,85,91^ More recent work shows that additive action can extend beyond primary nucleation to sustained solvent extraction, coordination competition, crystallization/passivation coupling, direct ordering of photoactive phases, and regulation of later-stage coarsening or grain-boundary transport.^24,26,27,33,34,51,104^ In FAPI specifically, nitroxide radicals such as TEMPO have recently been shown to modify phase stability and optoelectronic performance through subtle coordination and redox interactions.^63,70^ The present study focuses on a low-loading bulk-additive condition of only 1 mol% TEMPO, showing that even a small compositional perturbation can reorganize crystallization dynamics and the final microstructural landscape.^70^

Despite this progress, robust links between additive chemistry, crystallization dynamics, and final microstructure remain difficult to establish. Additive-induced effects are often subtle and spatially non-uniform, appearing as shifts in grain-size distributions, boundary irregularity, defect burden, or intragrain texture rather than as dramatic phase changes.^2,7,9,54,70,76^ Conventional probes such as X-ray diffraction, electron microscopy, and spectroscopic mapping remain indispensable, but they often sample limited regions or numbers of grains relative to the large-area heterogeneity of processed films.^9,30,55^ In rapidly processed absorbers, grain-boundary disorder and defect localization are especially relevant because they can couple directly to carrier recombination, transport losses, and device-to-device reproducibility.^2,7,13,54^ Resolving these non-equilibrium processing–structure relations increasingly requires per-grain, data-enabled approaches that convert large image populations into physically meaningful microstructural observables.^10,18,39,74,78,80,82,84,100^

Data-driven image analysis is increasingly well positioned to address this gap. Computer vision and segmentation tools can move bright-field microscopy from a qualitative inspection tool to a scalable route for inferring crystallization pathways, comparing kinetic landscapes, and connecting processing conditions to measurable microstructural outcomes under manufacturing conditions.^10,18,39,74,78,82,84,100^ Bright-field imaging is especially attractive here because it is non-destructive, experimentally simple, compatible with large fields of view, and readily scalable when coupled with robust segmentation tools.^70,80^ The scientific objective is materials-driven, but the image-analysis layer is essential rather than auxiliary.

Against this background, we investigate pristine FAPI and TEMPO-modified FAPI films processed by FIRA using a unified framework that links time-resolved crystallization videos, static bright-field micrographs, per-grain morphology, and derived kinetic descriptors. The analysis proceeds in three connected stages. First, a segmentation and quality-control pipeline isolates statistically large populations of physically meaningful spherulitic grains. Second, grain-level descriptors, including size, texture entropy, defect burden, radial structure, and crowding metrics, are extracted from the validated masks. Third, these descriptors are combined with direct video-derived transformed-fraction data to construct effective growth-rate distributions, transported kinetic reconstructions, and spatial signatures that capture how final film geometry reflects the underlying crystallization pathway.^6,14,19,73,81^ The resulting analysis reveals a clear contrast between pristine FAPI and FAPI–TEMPO: the additive delays and narrows the dominant crystallization burst, contracts the accessible kinetic landscape, and reduces the prevalence of optically defect-like outer-front heterogeneity while preserving the overall spherulitic growth motif.

## Results

### Image segmentation and grain curation

The analysis rests on a segmentation-based workflow that converts bright-field microscopy of rapidly crystallized perovskite films into curated grain instances and, ultimately, into quantitative descriptors of morphology, defect loading, spatial organization, and growth kinetics. This strategy is particularly well suited to FIRA-processed films, which form large, optically resolvable spherulites whose internal radial texture and mutual impingement patterns are clearly visible in bright-field imaging. Optical micrographs therefore contain rich structural information, but converting that information into robust, per-grain observables remains challenging when image quality, contrast, and local grain crowding vary across a dataset.^8,50,53^

**Fig. 1:**
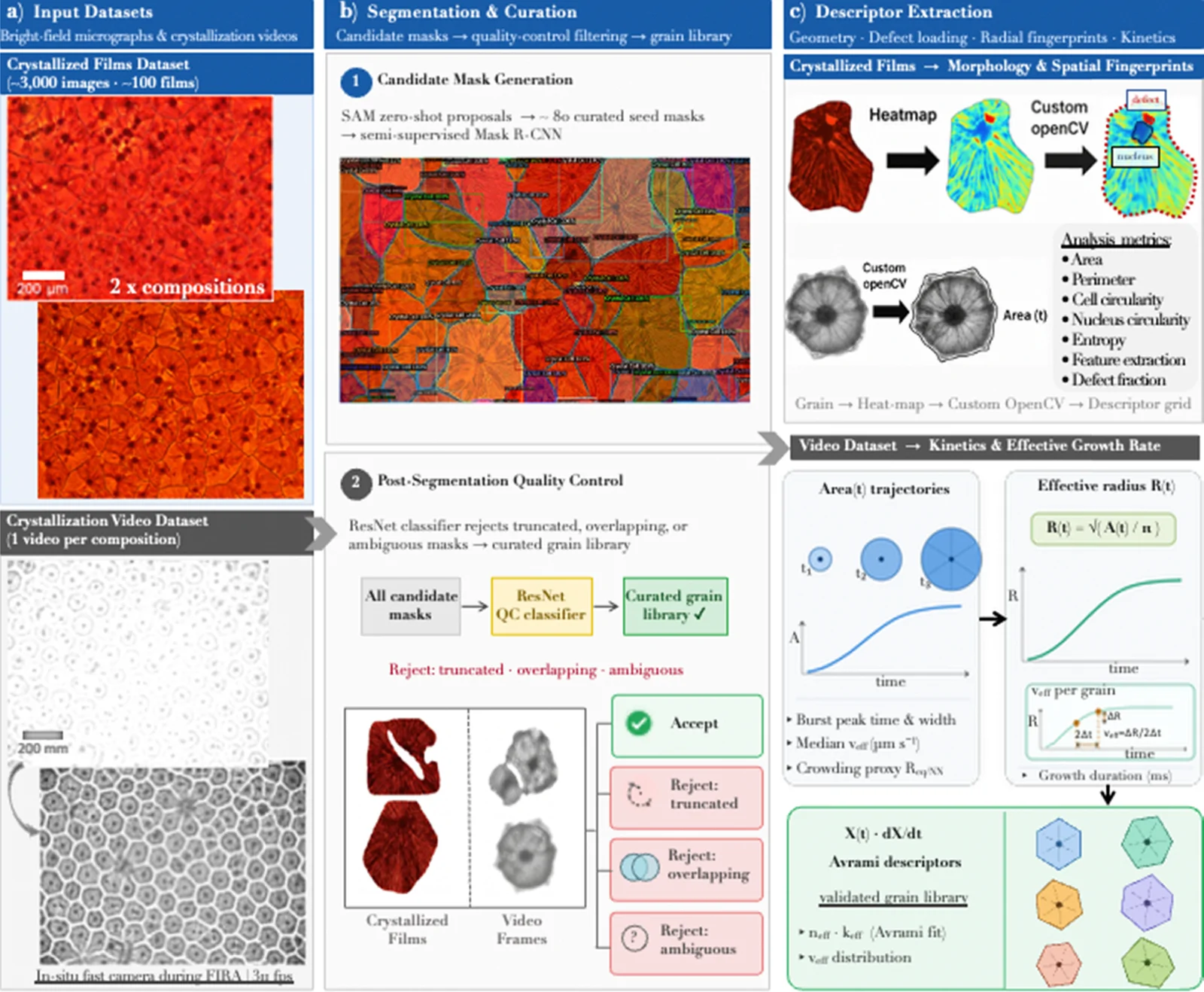
Segmentation-based workflow for per-grain analysis of FIRA-crystallized perovskite films. (a) Input datasets: a large static bright-field dataset from fully crystallized FAPI and FAPI–TEMPO films, and a complementary time-resolved dataset of *in situ* crystallization videos acquired during FIRA processing. (b) Segmentation and curation pipeline. Candidate masks are generated using SAM zero-shot proposals followed by semi-supervised Mask R-CNN refinement (1), then filtered through a ResNet-based quality-control stage (2) to reject truncated, overlapping, or ambiguous instances, yielding a curated grain library. (c) Descriptor extraction from validated grains. For crystallized films, custom heat-map and OpenCV-based representations quantify grain geometry, defect loading, and radial/spatial signatures. For the video dataset, tracked grain areas are converted into effective radius trajectories *R*(*t*) and related kinetic descriptors, including growth-rate proxies and Avrami-type parameters.

The full analysis pipeline is summarized in Fig. 1. It is organized into three connected stages. First, two complementary input datasets are assembled Fig. 1a. Second, these images are transformed into a curated grain library through segmentation and post-segmentation quality control Fig. 1b. Third, the validated grain instances are converted into descriptor-ready objects for morphology, spatial-signature mapping, and kinetics analysis Fig. 1c. Raw optical images are thus not analyzed directly at the pixel-field level, but are converted into physically interpretable single-grain objects that define a common basis for all downstream comparisons.

As shown in Fig. 1a, we used two distinct but complementary data streams. The first is a static dataset of fully crystallized films comprising 3088 bright-field micrographs (1544 per composition) collected from about 100 processed films across two compositions, pristine FAPI and FAPI–TEMPO. After segmentation and mask-quality control, these micrographs yield a curated library of 180 545 validated FAPI grains and 241 619 validated FAPI–TEMPO grains (422 164 in total), which provides the statistical basis for all grain-level distributions reported below. Because mask-quality control intentionally retains only high-confidence grain instances, this curated library is optimized for robust descriptor extraction rather than exhaustive enumeration of every crystallized object. These images capture the final microstructure with sufficient statistical depth to quantify grain morphology, radial structure, defect-related heterogeneity, and spatial crowding across a broad population. The second is a time-resolved video dataset consisting of one crystallization video per composition, recorded *in situ* during FIRA processing. These videos preserve the temporal evolution of individual spherulites and provide the direct information needed to reconstruct transformed-area trajectories, effective growth-rate proxies, and burst-like crystallization dynamics. The static and dynamic datasets therefore serve different but complementary roles: the static set establishes population-level end-state morphology, whereas the video preserves temporal fidelity to the crystallization event itself and serves as the kinetic anchor. Because only one video is available per composition, the video-derived burst and timing metrics are interpreted comparatively rather than as replicate-resolved population estimates; the internal consistency of the video analysis is documented by the frame-level quality-control diagnostics in Fig. S10–S13.

To make grain-level extraction robust to non-ideal imaging conditions, the segmentation stage in Fig. 1b was explicitly designed to favor robustness over purely automated throughput. In the first step Fig. 1b1, dense candidate masks were generated using a hybrid strategy combining zero-shot region proposals from the segment anything model (SAM) with semi-supervised instance refinement using a Mask R-CNN framework.^3,25,37^ Because no task-specific annotated dataset exists for this microscopy problem, SAM provides a practical initialization, while the refinement model, trained from approximately 80 manually curated seed masks, improves the physical consistency of mask boundaries for large spherulitic domains. This stage yielded an initial curated seed set spanning both crystallized-film images and representative video frames. More generally, segmentation and vision-foundation developments continue to expand the applicability of such pipelines to complex imaging problems, although the full pipeline here is tailored specifically to bright-field perovskite micrographs and was not transferred wholesale from another imaging domain.^4,101^

A subsequent quality-control stage Fig. 1b2 converts the initial mask population into a validated grain library. Here, a ResNet-based classifier was used to reject masks that were truncated by the image border, overlapped strongly with neighboring grains, or remained morphologically ambiguous. Because all subsequent geometry, radial, defect, and kinetics analyses depend directly on mask fidelity, this stage acts as a scientific selection criterion for defining the grain population admitted to downstream analysis. Accordingly, only masks corresponding to single, isolated, fully visible spherulites with closed boundaries and interpretable internal structure were retained. The output is therefore not simply a segmentation result, but a curated grain library that defines the common object set underlying the entire analysis.

Once validated masks are obtained, they are transformed into descriptor-ready representations Fig. 1c. For the static crystallized-film dataset, each grain is converted into custom heat-map and OpenCV-derived representations from which we extract grain area, perimeter, circularity distortion, nucleus circularity, the heat-map texture entropy (the reported whole-grain entropy, in bits per pixel) together with the companion intensity-based scalar entropies entropy(bits) and entropy_norm_(bits), defect fraction, and radial/spatial signatures that characterize internal texture and local heterogeneity. For the video dataset, each tracked grain yields an area trajectory, which is converted into an effective radius proxy. This representation is then used to quantify burst timing and width, growth duration, the direct video-derived median effective growth rate, crowding-sensitive proxies, and Avrami-type descriptors derived from transformed-fraction dynamics. This shared single-grain representation is what makes the FAPI *versus* FAPI–TEMPO comparison statistically well-founded while preserving the distinction between end-state morphology and kinetic evolution.

Supporting details of the computational implementation are provided in the SI. Fig. S1 describes the mask-to-descriptor conversion logic, while Fig. S2 and S3 summarize the classifier-based curation stage qualitatively and quantitatively. With this common per-grain basis established, we next examine how pristine FAPI and FAPI–TEMPO differ in their microstructural organization and crystallization behavior.

### Per-grain morphology and defect-aware descriptors

After segmentation and mask-quality filtering, only high-fidelity crystal domains are retained for quantitative analysis. These curated grains satisfy strict physical criteria, including a single nucleation center, a continuous boundary, sufficient internal contrast, and no truncation by the image border. All downstream morphology, defect, and kinetics-linked descriptors are therefore constructed from validated masks rather than from the full set of raw segmentation outputs. In practice, this converts the initial image collection into a physically curated grain library suitable for statistically meaningful comparison between pristine FAPI and FAPI–TEMPO.

Interpreting these grains requires that the optically segmented spherulites correspond to the photoactive α-FAPbI_3_ phase, which we establish at the film level for the films analysed here. X-ray diffraction is dominated by the α-FAPbI_3_ reflections, with the α(100) reflection at 2*θ* ≈ 14° clearly resolved and the full α series (≈24.3, 28.1, 31.5 and 40.3°) dominant over residual *δ*-FAPbI_3_/PbI_2_ markers; the two compositions show essentially identical peak positions, indicating no measurable lattice shift within the resolution of these measurements. Steady-state photoluminescence peaks at 811 nm (*E*_g_ ≈ 1.53 eV) for both compositions with no composition-dependent shift, consistent with photoactive α-FAPbI_3_ (Fig. S20 and S21; see SI, “Structural and optical phase validation”). The same FIRA process is further characterized structurally and at the device level in our previous work.^63,70^ Because the segmented spherulites are the dark, strongly absorbing domains in bright-field imaging, whereas *δ*-FAPbI_3_ is transparent and non-emissive, the morphology descriptors below are interpreted as describing predominantly α-FAPbI_3_-rich spherulitic domains rather than unvalidated optical contrast alone.

From each validated grain we extract a compact but physically interpretable set of geometric and information-theoretic descriptors, including area, perimeter, circularity distortion, and the heat-map texture entropy *h*_m_. Area and perimeter quantify grain scale and boundary extent, while circularity distortion measures deviation of the grain envelope from an ideal spherulitic disk. The heat-map texture entropy provides a label-free descriptor of internal optical disorder and texture complexity, distinguishing optically uniform grains from those with stronger internal heterogeneity. In parallel, optically defect-like regions and nucleus-related domains are extracted from per-grain heat-map representations using a custom OpenCV-based workflow, allowing each grain to be characterized not only by its outer shape but also by its internal structural organization. These descriptors were selected *a priori* from spherulitic and non-equilibrium growth physics rather than optimized for class separation; we therefore claim physical interpretability and non-redundancy—supported by the weak pairwise descriptor correlations in Fig. S4—rather than uniqueness or completeness. Throughout this work, the term defect refers to optically defined intragrain regions (defect-like inclusions and secondary-contrast domains identified from the heat-map representation), not to directly resolved crystallographic point defects; the phase identity of the crystallized domains is established above and in the corresponding SI validation figures.

**Fig. 2:**
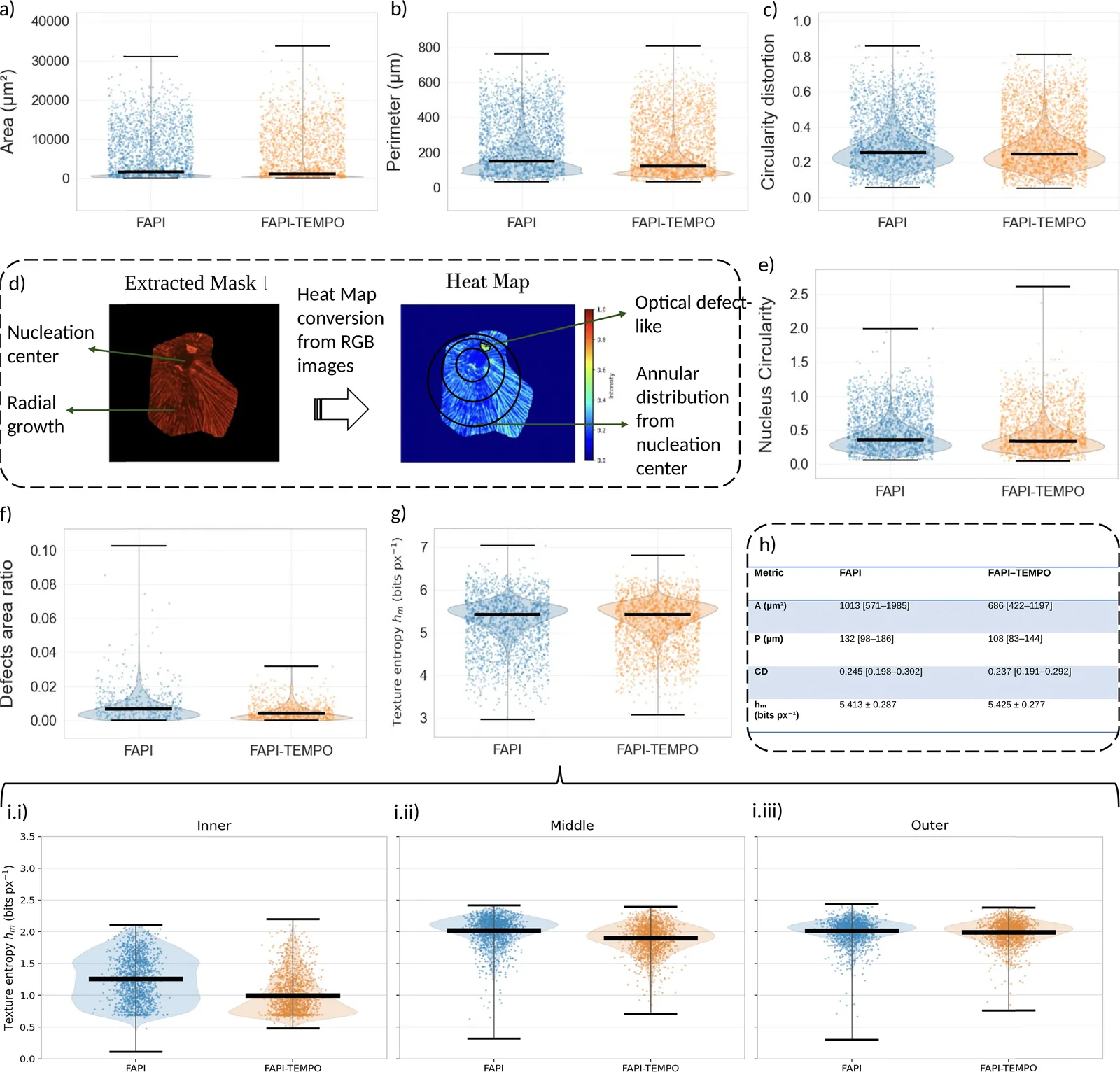
Grain-resolved morphology and defect-aware intragrain descriptors extracted from validated masks. (a)–(c) Grain area, perimeter, and circularity-distortion distributions for FAPI and FAPI–TEMPO. (d) Descriptor-extraction bridge from validated grain mask to heat-map representation, showing identification of the nucleation center, radial organization, segmented optical defect-like domains, and annular sampling relative to the grain center. (e)–(g) Comparisons of nucleus circularity, defect-area ratio, and heat-map texture entropy *h*_m_ in bits per pixel. (h) Embedded summary of the primary mask-derived scalar descriptors; area, perimeter, and circularity distortion are reported as grain-level median [interquartile range], while the texture-entropy row reports *h*_m_ as mean ± standard deviation in bits per pixel, matching panel (g). Table 1 reports the separate sample-level scalar statistics used for hypothesis testing. (i.i)–(i.iii) Heat-map texture entropy in inner, middle, and outer annuli, reported in bits per pixel; in each panel the thick bar denotes the mean and the whiskers span the minimum–maximum range across the per-micrograph values (1544 per composition).

Fig. 2 summarizes this transition from external grain geometry to defect-aware intragrain analysis. Panels (a)–(c) show the primary morphology distributions derived directly from the validated masks: grain area, perimeter, and circularity distortion. These descriptors already reveal a clear contrast between the two compositions: pristine FAPI forms larger grains with broader size distributions, whereas FAPI–TEMPO yields a smaller-grained and more morphologically constrained population. Panel (d) then illustrates the descriptor-extraction bridge from segmented grain to heat-map representation, showing how the nucleation center, segmented optical defect-like domains, and annular organization relative to the grain center are identified. This step is conceptually important because it links outer morphology to the intragrain fields used later for radial signature analysis.

Panels (e)–(g) extend the comparison beyond outer grain shape. Nucleus-related circularity in panel (e) probes the regularity of the central crystallization core after rapid FIRA growth, while the defect-area ratio in panel (f) quantifies defect loading and the heat-map texture entropy in panel (g) reports local optical texture complexity in bits per pixel. Panel (h) summarizes the same figure-level morphology descriptors, including *h*_m_ with the same units as panel (g), while Table 1 provides the separate sample-level scalar statistics used for hypothesis testing. Panels (i.i)–(i.iii) further partition the heat-map texture entropy into inner, middle, and outer annular zones (in bits per pixel), showing that disorder is not distributed uniformly across the grain radius and that the composition dependence is concentrated in the inner and middle annuli and converges at the outer growth front.

**Table 1:** Sample-level nonparametric comparison of the principal morphology and transported kinetics descriptors for FAPI and FAPI–TEMPO. Medians are reported with bootstrap 95% confidence intervals; Cliff's *δ* is the effect size

Descriptor	FAPI	FAPI–TEMPO	Δ median (FAPI − TEMPO)	Cliff's *δ*
Area median (µm^2^)	1039.15 [1011.51, 1066.74]	682.28 [669.73, 694.42]	356.87 [327.01, 386.93]	0.621
Perimeter median (µm)	133.51 [131.77, 135.22]	107.58 [106.57, 108.72]	25.93 [24.00, 27.91]	0.614
Equivalent radius median (µm)	18.19 [17.94, 18.43]	14.74 [14.60, 14.87]	3.45 [3.18, 3.73]	0.621
Transported *v*_eff_ median (µm s^−1^)	3.19 [3.15, 3.23]	2.59 [2.56, 2.61]	0.60 [0.56, 0.65]	0.660
Texture entropy *h*_m_ median (bits px^−1^)	5.516 [5.506, 5.525]	5.525 [5.520, 5.530]	−0.008 [−0.020, 0.002]	−0.006
Circularity-distortion median	0.2459 [0.2448, 0.2466]	0.2371 [0.2361, 0.2380]	0.0087 [0.0075, 0.0100]	0.293
Nucleus-circularity median	0.3382 [0.3345, 0.3407]	0.3158 [0.3133, 0.3180]	0.0224 [0.0178, 0.0261]	0.275
Defect-fraction mean	0.0048 [0.0045, 0.0050]	0.0026 [0.0024, 0.0028]	0.0022 [0.0019, 0.0025]	0.308

These descriptors define the static morphological backbone of the study. They are used first to compare the two compositions at the grain-population level and later to connect final-state morphology with kinetics-informed quantities and spatial signatures. The morphology section also includes a transported effective growth-rate proxy, reported at the sample level in Table 1; unlike the direct video-derived median reported later in Table 2, this quantity is obtained by mapping the broader static grain population onto the video-anchored effective time axis. Additional descriptor-level support is provided in the SI. Fig. S4 shows that grain-level correlations between the growth proxy *v*_R_, circularity distortion, defect fraction, and entropy remain weak overall, supporting the use of a multidimensional descriptor space rather than a single controlling variable. Fig. S5 and S6 further connect the main-text radial and crowding analyses to compact scalar summaries of radial defect localization and to grain-level crowding–kinetics trends.

**Table 2:** Kinetic summary descriptors extracted from video-derived and transported crystallization analysis

Quantity	Definition/source	FAPI	FAPI–TEMPO
Burst peak time (ms)	Time of the dominant maximum in video-derived d*X*/d*t*	46.0	172.0
Burst width (ms)	Equivalent burst-duration metric *w*_eq_ from video-derived d*X*/d*t*	43.24	16.64
Growth duration (ms)	Effective duration of the main crystallization event from the video-derived transformation curve	138.0	174.0
Median *v*_eff_ (µm s^−1^)	Direct video-derived grain-level effective growth-rate proxy from tracked grain areas	4.44	3.78
Transported *n*_eff_	Effective Avrami exponent from transported *X*(*t*) fit	0.879	0.827
Transported *k*_eff_	Effective Avrami rate parameter from transported *X*(*t*) fit	0.0912	0.1033
Median *R*_eq_/NN	Grain crowding/impingement proxy linked to the local growth environment	0.5666	0.5219

To test whether the composition-dependent shifts observed in Fig. 2 remain robust at the sample level, we performed nonparametric between-group comparisons using sample-level descriptor summaries, with each sample treated as an independent statistical unit. Median grain area, perimeter, equivalent radius, and the transported effective growth-rate proxy were all significantly larger in pristine FAPI than in FAPI–TEMPO, with large effect sizes according to Cliff's delta (*δ* = 0.61 to 0.66). Circularity distortion and nucleus circularity were also higher in FAPI, with smaller but systematic effect sizes (*δ* ≈ 0.27 to 0.29), while the mean defect fraction was modestly higher in FAPI (*δ* ≈ 0.31). By contrast, the whole-grain texture entropy *h*_m_ was statistically comparable between the two compositions (*δ* = −0.01, two-sided Mann–Whitney *p* = 0.77; companion intensity-based scalar entropies are reported in the SI). The whole-grain texture entropy is a single scalar over the full grain, whereas the annular analysis resolves where disorder sits along the grain radius; the two are complementary, so a comparable whole-grain value and a composition-dependent radial profile are consistent rather than conflicting. Resolved radially, the inter-composition difference is concentrated in the inner and middle annuli—where pristine FAPI carries higher texture entropy—and converges at the outer growth front, so that FAPI–TEMPO exhibits a steeper inner-to-outer radial gradient. These statistics indicate that TEMPO does not change the total amount of intragrain disorder, but redistributes it spatially while simultaneously narrowing the size and growth-rate landscape. The corresponding sample-level distributions and the integrated nonparametric statistical summary are shown in Fig. S7 and S8.

This static comparison also introduces the next section. If TEMPO regularizes the final grain population and narrows the accessible morphology space, the natural next question is whether the same contrast is visible in the time structure of crystallization itself.

### Crystallization kinetics from segmented grain masks

Starting from curated grain masks extracted from the two time-resolved FIRA videos, we reconstruct crystallization kinetics for pristine FAPI and FAPI–TEMPO at the grain level. The goal is to preserve a direct connection to the experimentally observed video evolution while building a morphology-informed representation that can be compared systematically between the two compositions and related back to the much larger static dataset. In this framework, the videos provide the kinetic anchor, whereas the static grain library provides the statistical depth needed to interpret the reconstructed curves within a descriptor-based framework. The morphology-transported kinetics introduced below use the video-derived transformation as a temporal anchor and map the larger static grain population onto the corresponding effective time axis, thereby extending the kinetic comparison beyond the limited number of directly observed events. A representative fast pyrometer trace acquired during FIRA is provided in the SI (Fig. S9) as contextual support for the transient thermal environment during annealing. In the present analysis, the thermal trace is used to illustrate the transient heating profile during the FIRA event, while crystallization timing is established from the video-derived observables.

For each grain *i*, the segmented area *A*_*i*_(*t*) is converted into an equivalent circular radius,1
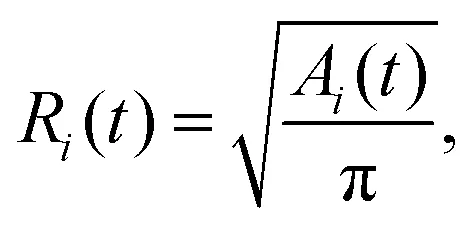
and an effective radial growth-rate proxy is estimated from the time derivative of *R*_*i*_(*t*).2
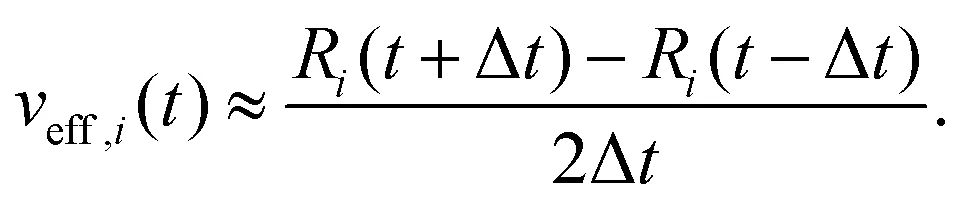


This construction does not assume perfectly circular growth. Rather, it provides a geometry-based comparative descriptor that maps the observed increase in segmented area onto an effective front-advance proxy with dimensions of length per time. At the film level, the same masks yield the transformed fraction *X*(*t*) = *A*_cryst_(*t*)/*A*_FOV_,3
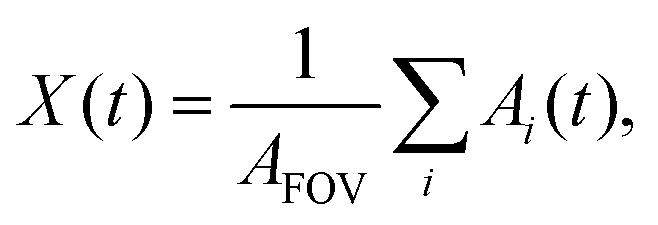
where *A*_FOV_ is the field-of-view area. Together, these definitions provide the bridge from discrete grain masks to kinetics-like observables that can be analyzed both directly in the measured videos and after morphology transport.

**Fig. 3:**
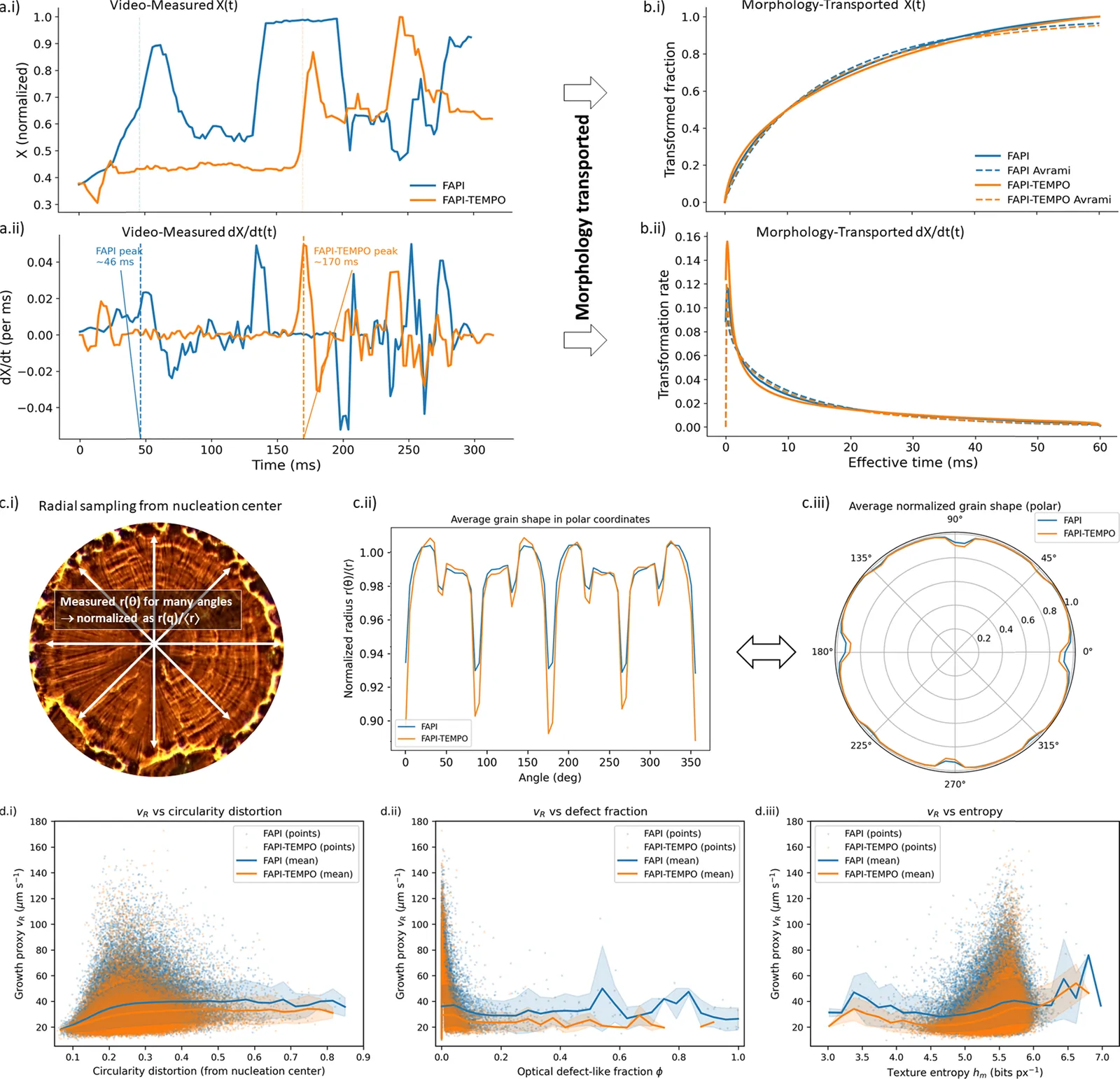
Video-derived kinetics, transported kinetics, polar grain-shape analysis, and grain-level morphology–kinetics relations for FAPI and FAPI–TEMPO. (a.i) Video-measured transformed fraction *X*(*t*) obtained directly from the time-resolved segmented grain areas within the field of view [eqn (3)]; this raw segmented-area transformation proxy is not constrained to be monotonic, whereas the monotonic-envelope representation used for the transported kinetic analysis is shown in Fig. S15 and S16. (a.ii) Corresponding video-measured transformation rate d*X*/d*t*, highlighting the dominant crystallization burst and its composition-dependent shift in timing. (b.i) Area-weighted transported transformation curve *X*(*t*) plotted *versus* effective time after geometry-based transport of per-grain area evolution; dashed curves show compact effective Avrami-type descriptors of the transported representation. (b.ii) Corresponding transported rate curve d*X*/d*t*(*t*). (c.i) Schematic of radial sampling from the nucleation center used to construct the normalized grain-boundary representation *r*(*θ*)/〈*r*〉. (c.ii) Composition-averaged normalized grain shape as a function of polar angle. (c.iii) The same averaged grain-envelope information shown in polar coordinates. (d.i)–(d.iii) Grain-level relations between the morphology-derived growth proxy *v*_R_ = *R*_eq_/*τ*_c_ and circularity distortion, optical defect-like fraction *φ*, and heat-map texture entropy *h*_m_, respectively; points denote individual grains and solid lines denote composition-specific mean trends.

Fig. 3 summarizes this bridge from direct observation to morphology-informed interpretation. Panels (a.i)–(a.ii) show the video-measured transformed fraction and transformation-rate curves obtained directly from the segmented crystallization movies. These traces remain as close as possible to the experimental signal and therefore define the direct kinetic anchor of the study. They show that the dominant crystallization burst occurs earlier in pristine FAPI, whereas FAPI–TEMPO remains delayed and then rises more sharply. This contrast is particularly clear in the traces, where the burst maxima differ both in timing and concentration, indicating that the additive does not simply suppress growth uniformly but reorganizes the temporal structure of the transformation.^23,30,41,51,58,91,99^

Panels (b.i)–(b.ii) show the transported representation, in which the video-derived kinetics are used as a temporal anchor and the larger static grain population provides the broader morphological statistics. Operationally, per-grain area evolution is mapped onto an effective-radius coordinate and then onto an effective time axis, allowing the dynamic contrast seen in the videos to be compared against the much larger static population. These transported curves are therefore not direct measurements of local kinetics, nor are they simply smoothed versions of the measured video traces. Rather, they are statistically extended, kinetics-like comparative descriptors that place the dynamic and static datasets on a common statistical footing. In this form, the contrast between FAPI and FAPI–TEMPO remains visible in a more compact and normalized way: pristine FAPI remains associated with a kinetically broader transformation pathway, whereas FAPI–TEMPO retains a delayed and more concentrated evolution. The dashed Avrami overlays in panels (b.i)–(b.ii) are included only as compact effective descriptors of the transported curves rather than as literal mechanistic fits, because FIRA crystallization is burst-like, non-isothermal, and spatially non-uniform.^6,14,19,73,81^

The effective Avrami exponents extracted from the transported curves (*n*_eff_ = 0.879 for FAPI and 0.827 for FAPI–TEMPO) are both below unity, a feature that is not straightforwardly interpretable in classical Avrami theory. In the present case, these sub-unity values are best understood as effective shape descriptors of a truncated, burst-like transformation observed through the morphology-transport window rather than as indicators of any literal nucleation dimensionality or elementary growth mechanism. Because FIRA crystallization is strongly non-isothermal, spatially non-uniform, and effectively sampled over a limited transformation interval, the fitted exponents mainly capture how sharply the transported curves rise and saturate under these constrained conditions. They are therefore retained only as compact comparative descriptors and are not assigned direct mechanistic meaning.^19,73^

Although the present dataset does not directly resolve precursor speciation or intermediate-phase composition, the observed delay and narrowing of the dominant crystallization burst in FAPI–TEMPO are consistent with additive-induced modification of the precursor-to-solid conversion pathway in the mixed DMF/DMSO solvent environment. In iodide-perovskite precursor systems, solvent coordination and additive chemistry are known to regulate intermediate-state stability, supersaturation buildup, and the synchronization of nucleation and growth. Within that framework, the kinetics contrast observed here is more consistent with a constrained coordination and intermediate-evolution landscape under TEMPO addition than with a simple uniform reduction in growth speed.^15,20,35,41,47,70,88,93,98,99^

The direct and transported kinetics are complementary rather than redundant. TEMPO delays the dominant burst and makes it narrower, but the total effective growth duration remains longer (Table 2) because the transformed-fraction trajectory extends over a broader low-rate tail before and after the dominant event. The additive therefore concentrates the main burst while still prolonging the overall conversion window.

Fig. 3 next connects these kinetic observables more explicitly to grain morphology. Panels (c.i)–(c.iii) introduce the polar representation used to characterize the average grain envelope relative to the nucleation center. The grain boundary is sampled over many angles and converted into the normalized radial function, providing the geometrical basis for the polar-anisotropy descriptor used later in the spatial-signature analysis. This quantity is distinct from the simpler scalar circularity-distortion metric reported in Table 1: the latter measures global deviation of the grain envelope from a disk, whereas resolves angular fluctuations of the normalized radial front. These panels show that both compositions retain an overall spherulitic architecture while still exhibiting systematic front-shape deviations from an ideal circle.

Finally, panels (d.i)–(d.iii) show how the growth-proxy landscape relates to selected static grain descriptors. The scatter plots compare the morphology-derived growth proxy *v*_R_ with circularity distortion, optical defect-like fraction, and heat-map texture entropy *h*_m_, while the overlaid mean trends summarize the average dependence for each composition. Here *v*_R_ denotes the equivalent-radius growth proxy evaluated over the full static grain population (final equivalent radius normalised by the crystallization window *τ*_c_ = 640 ms); it is reported in µm s^−1^ and is distinct from the video-anchored kinetic rate of eqn (2) and Tables 1 and 2. Together, these panels show that the growth response is distributed over a broad multidimensional descriptor space rather than controlled by any single scalar variable. FAPI and FAPI–TEMPO occupy overlapping but distinguishable clouds, indicating that additive-induced changes in kinetics are reflected statistically in morphology space even though no single descriptor alone explains the full breadth of the growth-proxy distribution.

Fig. 3 therefore brings together four complementary views of the crystallization pathway: direct video kinetics, transported kinetics, polar front-shape characterization, and descriptor-conditioned growth-rate trends. Table 2 condenses the principal quantitative outputs, showing that the contrast between pristine FAPI and FAPI–TEMPO appears not only in burst timing, but also in effective growth metrics, crowding-related descriptors, and transported-fit parameters.

This kinetics reconstruction establishes the second major pillar of the analysis. The validated grain masks are not only a source of static morphology descriptors, but also the basis for a per-grain and morphology-informed description of transformation dynamics. We next ask where, within the final grains, the signatures of that non-equilibrium growth pathway are most strongly stored.

Supporting diagnostics, including the representative pyrometer trace (Fig. S9), transformed-fraction quality control (Fig. S10), burst-window annotations (Fig. S11), event-time histograms (Fig. S12), local Avrami reconstructions (Fig. S13), transport schematics (Fig. S14), weighting robustness (Fig. S15 and S16), and transported Avrami fits (Fig. S17), are provided in the SI.

### Out-of-equilibrium microstructural signatures

While Avrami-type fits compress the transformation into a few effective kinetic parameters, device-relevant behavior is ultimately governed by the spatial organization of the final microstructure. The central result of this section is that the strongest signature of the non-equilibrium growth pathway is stored near the grain periphery rather than uniformly across the grain interior. We therefore complement the grain-level kinetic analysis with a set of segmentation-derived microstructural signatures that retain information on front stability, internal texture, optical-heterogeneity accumulation, local crowding, and spatially correlated kinetic heterogeneity.

**Fig. 4:**
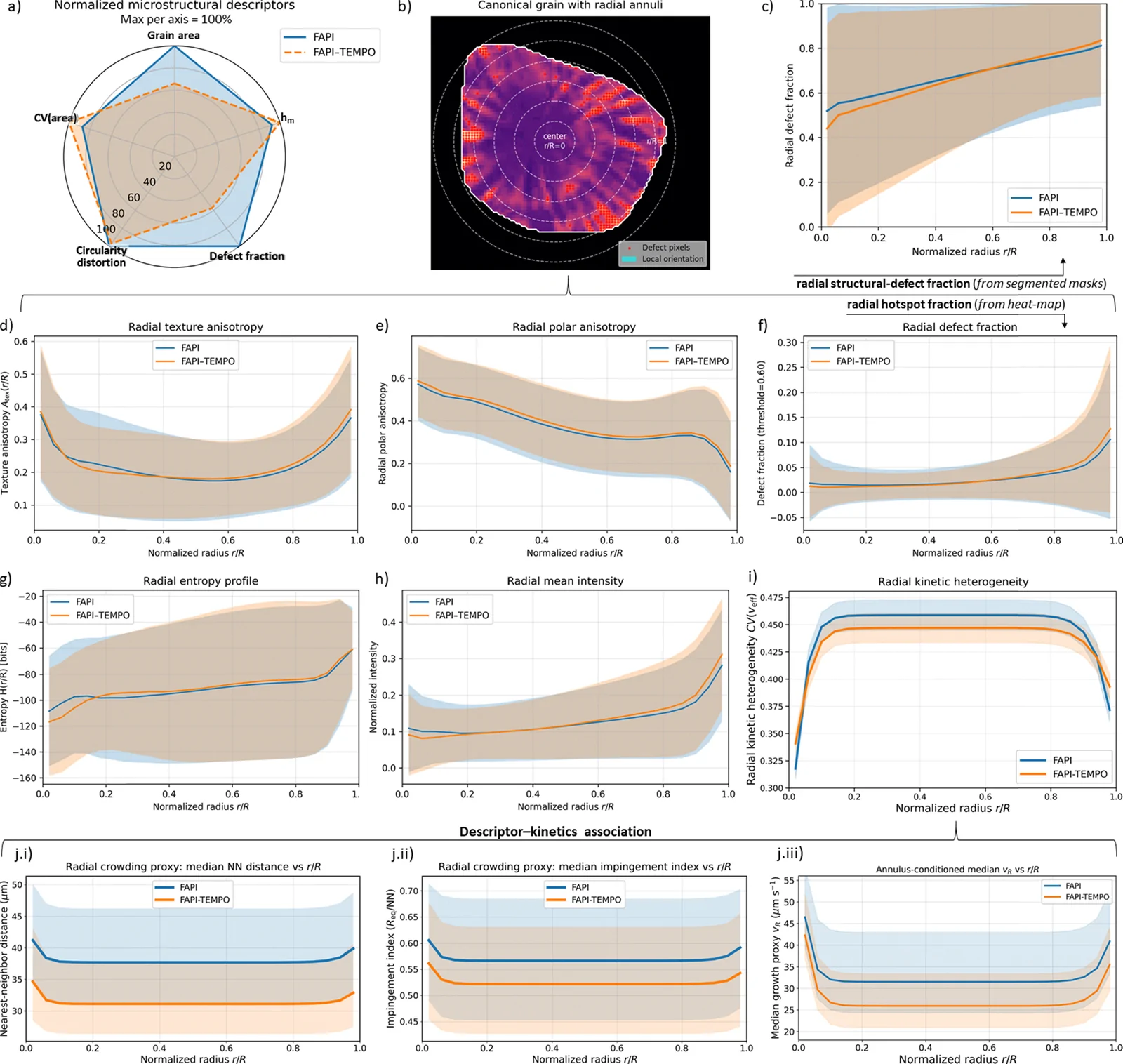
Segmentation-based microstructural signatures and descriptor–kinetics associations. (a) Radar chart of normalized film-level microstructural descriptors, summarizing the relative balance of grain area, area dispersion, circularity distortion, entropy, and optical defect-like fraction between FAPI and FAPI–TEMPO. (b) Canonical-grain construction used for radial averaging, illustrating the mapping of a segmented grain onto normalized annuli referenced to the grain center and boundary. (c) Radial optical defect-like fraction from segmented masks together with radial hotspot fraction from the heat-map representation. (d) Radial texture anisotropy *A*_tex_(*r*/*R*_eq_). (e) Radial polar anisotropy *A*_polar_(*r*/*R*_eq_). (f) Radial defect fraction *φ*(*r*/*R*_eq_). (g) Radial heat-map entropy contrast Δ*H*(*r*/*R*_eq_), a signed relative texture descriptor (not a positive-definite Shannon entropy; values may be negative). (h) Radial mean heat-map intensity. (i) Radial kinetic-heterogeneity proxy, expressed as CV(v_R_)(*r*/*R*_eq_). (j.i) Median nearest-neighbour distance *versus r*/*R*_eq_. (j.ii) Median impingement index *R*_eq_/NN *versus r*/*R*_eq_. (j.iii) Annulus-conditioned median growth proxy *ṽ*_R_(*r*/*R*_eq_). In panels (c)–(j), solid lines denote composition-averaged profiles and shaded bands indicate the spread across validated grains; for fraction-valued descriptors these spread bands may extend beyond the physical range [0,1] where the grain-to-grain spread is large. Additional radial quality-control and local-field support are provided in Fig. S18 and S19.

The descriptor framework used in this section is defined at the film level in Table 3 and visualized in Fig. 4(a). These quantities are directly extractable from bright-field micrographs, heat maps, and curated masks while retaining clear physical interpretation. Shape anisotropy and texture anisotropy describe boundary irregularity and directional ordering of intragrain optical texture, multiscale entropy contrast captures internal optical disorder, defect fraction quantifies the overlap between grains and optically defect-like regions, and measures the breadth of the grain-level growth-proxy landscape. Together, these descriptors define a compact but multidimensional descriptor space in which the two compositions can be compared without collapsing the dataset into a single scalar response.

**Table 3:** Microstructural descriptors extracted from bright-field micrographs, heat maps, and curated masks

Descriptor	Definition	Physical meaning/linked technique	Values (FAPI/FAPI–TEMPO)
*A*_polar_	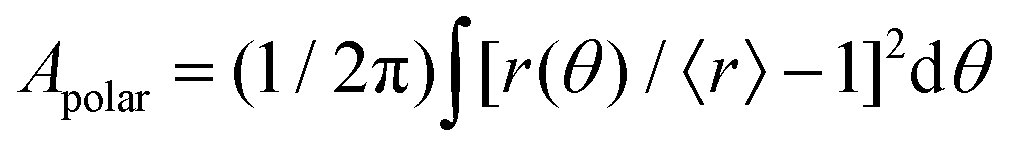	Angularly resolved front-shape anisotropy; deviation of the normalized grain envelope from circularity.	0.051 ± 0.042 0.052 ± 0.040
*A*_tex_	*A*_tex_ = |Σ|∇*I*|_p_*e*^(i2*θ*_p_)^|/Σ|∇*I*|_p_	Degree of directional ordering of intragrain optical texture.	0.1726 ± 0.0857 0.1646 ± 0.0728
Δ*H*	Δ*H* = *H*_0_ − *H*_*σ*_	Separates fine-scale from coarse-scale optical disorder.	0.272 ± 0.194 0.74 ± 0.18
*ϕ*	*ϕ* = *A*_def_/*A*_grain_	Fraction of grain area intersected by defect masks.	0.00659 ± 0.0273 0.0038 ± 0.0162
CV(*v*_R_)	CV(*v*_R_) = *σ*(*v*_R_)/〈*v*_R_〉	Dispersion of the grain-level effective growth-rate distribution.	0.4587 ± 0.0010 0.4470 ± 0.0013

The remainder of Fig. 4 resolves this descriptor space spatially. Panel (b) introduces the canonical-grain construction, in which segmented grains are mapped onto normalized annuli referenced to the grain center so that grains of different absolute size can be compared on the common coordinate. This transformation enables meaningful averaging of disorder, anisotropy, defect loading, and kinetics-linked observables across structurally comparable grains.

Panels (c)–(h) show that the outer grain regions carry a disproportionate share of the non-equilibrium microstructural signature. Optical defect-like fraction and hotspot fraction both increase toward the periphery, indicating that branching, impingement, and optically defect-like activity accumulate at the advancing front. Texture anisotropy and polar anisotropy are likewise radially structured, demonstrating that neither internal optical texture nor front stability is spatially uniform. Defect fraction, entropy, and mean heat-map intensity all increase toward the outer annuli, reinforcing that the grain edge is not only geometrically less regular but also optically and structurally more heterogeneous than the interior.

The kinetics-linked radial descriptor in Fig. 4(i) extends this picture into a spatially correlated growth-rate dispersion field. This quantity should be interpreted as an annulus-conditioned proxy for growth-rate heterogeneity rather than as a pixelwise local kinetics field. The radial kinetic-heterogeneity proxy remains consistently higher in FAPI than in FAPI–TEMPO, indicating that the additive narrows the accessible growth-rate landscape both globally and locally within the grain, consistent with the more synchronized pathway seen in the kinetics.

Panels (j.i)–(j.iii) reconnect this internal radial structure to the external growth environment. The median nearest-neighbour distance, impingement index, and annulus-conditioned median effective growth rate together show that FAPI–TEMPO evolves within a more constrained spatial neighbourhood. These trends indicate that the additive modifies both internal grain structure and the collective spatial environment of growth, linking local crowding to the observed narrowing of the kinetic landscape.

Taken together, Table 3 and Fig. 4 establish the final-state microstructure as a readable signature of the FIRA growth pathway. The combined radial, defect, and crowding descriptors show that TEMPO does not simply slow crystallization uniformly. Instead, it delays and narrows the dominant burst, suppresses optical-heterogeneity accumulation at the outer front, reduces grain-scale disorder and size dispersion, and narrows the accessible kinetic landscape while preserving the overall spherulitic growth motif. At the same time, the entropy results indicate that this regularization is not spatially uniform: in the annular analysis pristine FAPI carries higher texture entropy in the inner and middle (interior) zones, with the two compositions converging at the outer growth front, whereas the whole-grain texture entropy is comparable between compositions, consistent with a spatial redistribution rather than a change in the total amount of intragrain disorder.

Our imaging-derived results are consistent with additive-mediated modification of the precursor-to-solid conversion pathway in the mixed DMF/DMSO solvent system, where coordination and intermediate-phase evolution regulate crystallization timing and spatial organization.^20,35,70,88,93,98^ The radial and crowding descriptors show that these differences are stored most strongly near the grain periphery, where front instability, impingement, and optical-heterogeneity accumulation are most pronounced. Representative local distance-field maps shown in Fig. S19 further indicate that the framework can be extended toward more spatially resolved structure–property analysis beyond the grain-averaged and annular descriptors emphasized here.

## Summary

This work establishes a segmentation-based bright-field microscopy framework for quantifying how rapid FIRA processing imprints crystallization history into the final microstructure of halide-perovskite films. By combining time-resolved crystallization videos with a much larger static image population, the analysis resolves grain morphology, effective growth descriptors, and spatial signatures on a common per-grain basis. Applied to pristine FAPI and FAPI–TEMPO, the framework shows that even a low-loading additive perturbs crystallization in a systematic and measurable way: TEMPO delays and narrows the dominant crystallization burst, reduces grain-size dispersion, contracts the accessible effective-growth landscape, and suppresses optically defect-like activity at the outer growth front, while preserving the overall spherulitic growth class.

A key outcome is that the final film does not simply reflect whether crystallization was “faster” or “slower,” but how the full non-equilibrium pathway was reorganized in time and space. The combined morphology, annular entropy, defect, anisotropy, and crowding analyses show that TEMPO does not uniformly remove heterogeneity; rather, the data are consistent with a spatial redistribution of intragrain disorder, suppressing optically defect-like outer-front heterogeneity and lowering interior texture entropy while leaving the whole-grain texture entropy comparable. The additive therefore acts less as a simple growth suppressant than as a pathway selector that narrows the range of accessible crystallization trajectories under rapid annealing.^20,35,51,70,88,93,98^

This distinction matters for device optimization. In rapidly processed perovskite absorbers, grain-size statistics, front stability, defect localization, and local crowding are all expected to influence the balance between carrier transport, non-radiative recombination, and process reproducibility at the film level.^2,7,40,63^ The present results therefore suggest that additive-mediated control of transient crystallization can be evaluated not only through end-point device metrics, but also through image-derived microstructural signatures that report how a given process reshapes the accessible film-formation landscape before that information is obscured by the averaging inherent in device-level metrics. In this sense, the workflow provides a practical bridge between manufacturing-relevant processing, crystallization science, and device-relevant film quality.

## Methods

### Film preparation

FAPI and FAPI–TEMPO films were prepared using the same substrate and deposition protocol up to completion of the perovskite layer. Fluorine-doped tin oxide (FTO)-coated glass substrates (Pilkington NSG TEC) were cleaned using a 2% Hellmanex solution in water, followed by 30 min sonication in the same 2% Hellmanex solution, 15 min sonication in isopropanol, and 5 min oxygen-plasma treatment. A compact TiO_2_ layer (≈30 nm) was then deposited by spray pyrolysis at 450 °C from a 0.1 M precursor solution of titanium diisopropoxide bis(acetylacetonate) in anhydrous ethanol and acetylacetone. After deposition, the substrates were held at 450 °C for 5 min and then cooled to room temperature.

A mesoporous TiO_2_ layer was subsequently deposited by spin coating a diluted TiO_2_ paste (Dyesol 30 NR-D, 75 mg mL^−1^ in ethanol; 30 nm particle size) for 10 s at 4000 rpm with a 2000 rpm ramp, yielding a thickness of approximately 150–200 nm. The films were dried at 100 °C for 10 min and then annealed at 450 °C for 30 min under dry air to crystallize the mesoporous TiO_2_ scaffold.

The perovskite precursor solution consisted of FAI and PbI_2_ (1.5 M total concentration) dissolved in anhydrous DMF/DMSO (3 : 1, v/v). For the FAPI–TEMPO condition, TEMPO was added to the precursor at 1 mol%; the pristine FAPI precursor was prepared identically but without TEMPO. Perovskite films were deposited in a single spin-coating step at 4000 rpm for 10 s and crystallized by FIRA using a 640 ms IR pulse. Immediately after the pulse, the substrates were removed from the FIRA chamber and placed on a hotplate at 100 °C for 5 min to complete solvent removal. FIRA processing was carried out in a glovebox under N_2_ atmosphere.

### Data acquisition and image preprocessing

Bright-field optical microscopy was used to characterize crystallization in FIRA-processed FAPbI_3_ films with and without TEMPO additive. Two complementary datasets were analyzed: (i) one time-resolved crystallization video per composition, used as the direct kinetic anchor, and (ii) a larger static micrograph dataset, used to extract per-grain morphology and spatial-signature statistics with higher population-level support. The two original *in situ* crystallization sequences are provided as SI Movies SM1 and SM2 at https://doi.org/10.5281/zenodo.19681060.

Time-resolved crystallization videos were acquired using a dedicated *in situ* high-speed imaging setup built to monitor FIRA-induced crystallization during annealing. The system consisted of a XIMEA CB160MG-LX-X8G3 monochrome camera mounted on a Mitutoyo linear microscope positioned directly above the FIRA stage, enabling real-time video acquisition during the flash infrared annealing process. This home-built configuration was specifically designed to allow optical monitoring under FIRA conditions, with image contrast generated under the FIRA illumination conditions during the annealing event.

To monitor the transient thermal history during FIRA, an auxiliary fast infrared pyrometer (thermoMETER CT, model CTF-SF25) was positioned above the sample and focused on the top surface of the perovskite film, while the infrared irradiation was applied from the substrate side. The CTF-SF25 operates in the 8–14 µm spectral range, provides a 25 : 1 optical resolution, and has a nominal response time of 6 ms (95% signal). To place the pyrometric reading on a physical footing, the pyrometer was operationally calibrated against a direct contact reference: a fine-gauge (50 µm) K-type thermocouple with a digital readout was bonded to the FTO/glass substrate and recorded the substrate temperature under a 1 s calibration pulse comparable to and bracketing the 640 ms pulse used for film processing. The emissivity was set to *ε* = 0.90, which provided an operational match to the contact reference under this configuration. The resulting trace is therefore used as an approximate substrate-surface thermal history and cooling diagnostic rather than an absolute film-temperature measurement; crystallization timing was independently established from the video-derived observables, and the corresponding trace is provided in Fig. S9.

The camera provides a sensor resolution of 4704 × 3424 pixels (16.1 MP), a nominal frame rate of 311 fps, and a pixel size of 3.9 µm. The resulting image sequences were used to resolve the temporal evolution of nucleation, radial grain expansion, grain impingement, and the film-level transformed fraction throughout crystallization. This *in situ* video dataset provided the direct experimental kinetic anchor for the analysis, enabling extraction of per-grain area trajectories, effective growth-rate proxies, burst timing metrics, and transformation-rate curves for comparison between pristine FAPI and FAPI–TEMPO films.

For the large-area static imaging workflow, bright-field micrographs were acquired on an Olympus BX3M microscope equipped with an Olympus MPLN5X-1-7 objective and white-LED illumination (OMICRON LEDMOD). Images were recorded with a Hamamatsu ORCA-FLASH 4.0 LT CMOS camera at a resolution of 2048 × 2048 pixels. Under these conditions, the field of view at 5× was approximately 2.4 × 2.4 mm, corresponding to an approximate spatial sampling of 1.17 µm pixel^−1^. To enable high-throughput acquisition, the microscope was coupled to a Thorlabs MLS203-1 XYZ scanning stage controlled through a custom Python-based microdevice manager.

Prior to segmentation and descriptor extraction, images were preprocessed to ensure compatibility with the downstream analysis workflow. Preprocessing included format conversion, contrast standardization, consistency checks across fields of view, and removal of border or acquisition artefacts when required while preserving complete grain morphology within the analyzed region. Time-resolved video sequences were used to extract crystallization trajectories, whereas the static image library provided the broader statistical basis for morphology distributions, radial signatures, and crowding metrics. Additional details on data organization, annotation format, and preprocessing are provided in SI Section S1.

### Segmentation and grain validation

Grain masks were obtained through a segmentation-based workflow combining zero-shot proposal generation, supervised refinement, and post-segmentation quality control. Candidate masks were first proposed using the segment anything model (SAM) in zero-shot mode. These proposals were then refined using a Detectron2-based instance-segmentation model trained on manually curated examples of physically meaningful spherulitic grains. A subsequent ResNet-based binary classifier was used to reject faulty masks, including truncated grains, overlaps, ambiguous boundaries, and segmentation artefacts. Only validated grains were retained for quantitative analysis. Full details of the segmentation workflow, manual curation criteria, classifier design, and representative quality-control examples are given in SI Section S2 and Fig. S1–S3.

### Grain descriptors and spatial-signature extraction

For each validated grain, geometric and image-derived descriptors were extracted from the grain mask and the corresponding bright-field micrograph. These included grain area, perimeter, equivalent radius, circularity-related shape measures, the heat-map texture entropy and companion intensity entropies, defect fraction, and anisotropy descriptors characterizing both boundary irregularity and intragrain optical texture. The equivalent radius was defined as
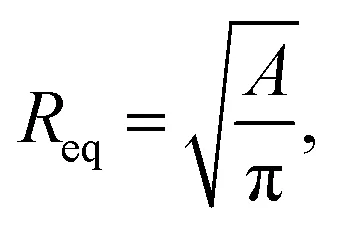
where is the grain area.

To compare grains of different size on a common basis, intragrain quantities were additionally mapped onto a normalized radial coordinate. This canonical representation enabled the construction of radial signatures for disorder, defect loading, anisotropy, crowding-related observables, and kinetic heterogeneity, allowing direct comparison of grain interiors and outer growth fronts across the population. Local distance-field maps, used in Fig. S19 to visualize more spatially resolved structure–property organization around validated grains, were generated from the same canonicalized mask representations and analyzed separately from the grain-averaged and annular descriptors emphasized in the main text. Full descriptor definitions, extraction details, radial-analysis procedures, and local distance-field diagnostics are provided in SI Sections S2 and S7, as well as Fig. S4–S6 and S18–S19.

### Video-based and morphology-transported kinetics analysis

Time-resolved crystallization videos were segmented frame by frame to extract film-level and grain-level kinetic observables. The transformed fraction, its derivative, event-count proxies, and local Avrami-type diagnostics were obtained from the video-derived masks and used to identify burst timing, width, and synchrony differences between FAPI and FAPI–TEMPO. From these observables we also extracted compact scalar kinetic summary descriptors, including burst peak time, burst width, growth duration, effective growth-rate statistics, and effective transported-fit parameters reported in the main text. Grain-level area trajectories were converted to equivalent-radius trajectories, from which an effective growth-rate proxy was estimated. These video-derived measurements provided the direct kinetic anchor for the study. Details of the video-based kinetic extraction, the representative pyrometer trace, and the associated quality-control analysis are given in SI Section S5 and Fig. S9–S13.

Because only one crystallization video was available per composition, these direct measurements were complemented by a transported kinetics framework in which the larger static grain population was mapped onto an effective time axis anchored by the video-derived kinetics. This enabled reconstruction of kinetics-like curves from the static morphology distributions and allowed the experimentally observed crystallization pathways to be linked to the broader microstructural dataset. Accordingly, video-derived burst and timing metrics are interpreted comparatively rather than as replicated population estimates. Weighting robustness and transported-kinetics diagnostics are presented in SI Section S6 and Fig. S14–S17.

### Statistical analysis

Unless otherwise stated, scalar descriptors are reported as mean standard deviation across the validated grain population for each composition. Radial curves were computed grain-by-grain in normalized coordinates and then averaged across the ensemble; the shaded bands shown in the radial plots represent the spread across grains at each normalized radius. For growth-rate dispersion metrics such as
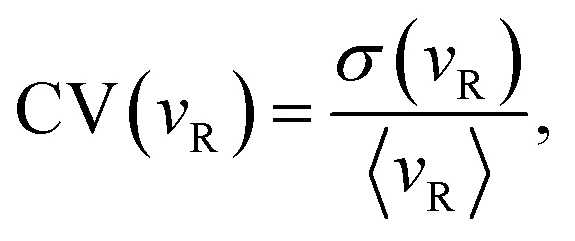
the reported uncertainty was estimated by bootstrap resampling of the grain-level population.

For the principal between-composition comparisons, descriptor values were additionally aggregated at the sample level—here, each sample is an individual micrograph (field of view; 1544 per composition, acquired across the 100 processed films)—and each sample was then treated as an independent statistical unit. Because several descriptor distributions were non-Gaussian and, in some cases, zero-inflated, between-group comparisons were performed using two-sided Mann–Whitney *U* tests. Effect sizes were quantified using Cliff's delta, with positive values indicating larger descriptor values in FAPI and negative values indicating larger values in FAPI–TEMPO. Confidence intervals for sample-level medians and median differences were estimated by bootstrap resampling (95% confidence intervals, percentile method). Multiple-testing correction across the selected descriptor set was performed using the Benjamini–Hochberg false-discovery-rate procedure. The whole-grain entropy reported in the main text is the heat-map texture entropy (in bits per pixel); the intensity-based scalar entropies entropy(bits) and entropy_norm_(bits) are retained in the dataset and reported in the SI as companion descriptors. The annular entropy analysis resolves the radial distribution of texture entropy within the grain and is reported in raw bits per pixel. Supplementary figures provide additional robustness checks, descriptor-level comparisons, and quality-control diagnostics.

## Conflicts of interest

There are no conflicts of interest to declare.

## Supplementary Material

MH-013-D6MH00916F-s001

## Data Availability

The two time-resolved *in situ* crystallization videos analysed in this study for pristine FAPI and FAPI–TEMPO are available as supplementary information (SI) Movies SM1 and SM2 and are deposited at Zenodo: https://doi.org/10.5281/zenodo.19681060. The analysis code used for segmentation-assisted grain extraction, morphology quantification, kinetics reconstruction, and statistical analysis is publicly available at https://github.com/sandysanche-glitch/fira-fapi-kinetics. Processed data supporting the figures and tables are included in the SI. Supplementary information is available. See DOI: https://doi.org/10.1039/d6mh00916f. The full raw static microscopy image dataset is not deposited in a public repository because of its size, but it is available from the corresponding author upon reasonable request.
