# Supplementary material for: Non-equilibrium crystallization and spatial fingerprints in flash IR-annealed halide perovskite films
Source: Mater Horiz. 2026 Jul 17. Online ahead of print. doi: 10.1039/d6mh00916f (PMC13394456; doi:10.1039/d6mh00916f)
Supplement: MH-OLF-D6MH00916F-s001 [file MH-OLF-D6MH00916F-s001.pdf]

Supplementary Information (SI) for Materials Horizons.
This journal is © The Royal Society of Chemistry 2026

SUPPLEMENTAL INFORMATION

Non-equilibrium crystallization and spatial fingerprints in flash IR-annealed halide perovskite films

Ornella Vaccarelli[1], Tiziano Agostino Caldara[2], Christophe Gisler[1], Jean Hennebert[1], Sandy Sánchez Alonso[3,2]

[1]Institute of AI and Complex Systems (iCoSys), School of Engineering and Architecture of Fribourg (HEIA-FR), University of Applied Sciences and Arts Western Switzerland (HES-SO), 1700 Fribourg, Switzerland

[2]Laboratory of Photonics and Interfaces, Institute of Chemistry and Chemical Engineering, École Polytechnique Fédérale de Lausanne, 1015 Lausanne, Switzerland

[3]Institute of Smart and Secured Systems (iSIS), School of Engineering and Architecture of Fribourg (HEIA-FR), University of Applied Sciences and Arts Western Switzerland (HES-SO), 1700 Fribourg, Switzerland

E-mail: sandy.sanchezalonso@epfl.ch

This Supplemental Information provides the methodological details, quality-control analyses, descriptor-level statistics, and literature-positioning elements supporting the main text. It documents: (i) segmentation-based extraction of per-grain descriptors from bright-field microscopy, (ii) structural and optical validation that the segmented domains are α-$FAPbI_3$, (iii) video-measured kinetics and burst metrics, (iv) the transported-kinetics bridge used to map static morphology distributions onto an effective time axis, (v) grain-scale and radially resolved microstructural signatures, (vi) representative true local pixelwise distance-field analyses, (vii) sample-level nonparametric statistical comparisons between FAPI and FAPI–TEMPO, and (viii) a benchmarking perspective situating the present descriptor framework relative to recent advanced studies of perovskite crystallization kinetics and process physics. The two time-resolved in situ crystallization videos analysed in this work are additionally provided as Supplementary Movie SM1 (pristine FAPI) and Supplementary Movie SM2 (FAPI–TEMPO). A curated public repository containing the analysis scripts, processed CSV outputs, and figure-support files used in this study is available in the GitHub repository.

1 Input data, file organization, and segmentation format

1.1 Datasets

Two compositions are analyzed: pristine $FAPbI_3$ (FAPI) and FAPI–TEMPO. Two data streams are used:

1. **Time-resolved video dataset (one per composition).** Bright-field micrographs acquired during flash infrared annealing (FIRA) are segmented frame by frame to yield time-

resolved grain masks. These data are used to extract the video-measured kinetics $n(t)$, dn/dt, $X(t)$, and dX/dt, together with the associated burst metrics. The corresponding raw crystallization sequences are supplied as Supplementary Movie SM1 (FAPI) and Supplementary Movie SM2 (FAPI–TEMPO).

2. **Large static datasets.** End-state segmentation outputs (grain masks, defect masks, and image-derived fields) are used to extract morphology distributions and spatial signatures with stronger statistical support. The static dataset comprises approximately 3000 bright-field micrographs (1544 per composition); after segmentation and ResNet-based mask-quality control, it yields a curated library of 180,545 validated FAPI grains and 241,619 validated FAPI–TEMPO grains (422,164 in total). The statistical unit used for the non-parametric comparisons is the micrograph; the per-micrograph grain-count medians are 119 for FAPI and 162 for FAPI–TEMPO. These datasets are linked to the single-video kinetics through the transported-kinetics bridge.

1.2 Structural and optical phase validation

The descriptor framework is material-agnostic, so its physical relevance here depends on the segmented spherulites being the photoactive α-$FAPbI_3$ phase rather than residual intermediates, δ-$FAPbI_3$, or PbI_2-rich regions. For the present FAPI and FAPI–TEMPO films, processed with a single 640 ms FIRA pulse (with 1 mol% TEMPO for the additive condition), fresh X-ray diffraction and steady-state photoluminescence measurements were used as direct validation anchors. The XRD patterns are dominated by the α-$FAPbI_3$ perovskite reflections, with the $\alpha(100)$ reflection at $2\theta \approx 14^\circ$ and the higher-angle α series resolved for both compositions, while residual δ-$FAPbI_3/PbI_2$ signatures remain minor (Fig. SI20). The PL spectra peak near 811 nm for both compositions, with no composition-dependent shift, consistent with the photoactive α-$FAPbI_3$ absorber phase (Fig. SI21). The same FIRA process is further characterized structurally and at device level in our previous work on FIRA-crystallized FAPI (see main-text references). Accordingly, the optically segmented grains are identified as predominantly α-$FAPbI_3$ domains, and the morphology, optical-heterogeneity, and radial descriptors reported throughout this Supplement characterize predominantly α-$FAPbI_3$-rich spherulitic domains rather than unvalidated optical contrast alone.

1.3 JSON mask format and categories

Instance-level grain, nucleus, and defect annotations are exported as JSON files in a COCO-inspired format.[1, 2] In the present dataset, each JSON file is typically stored as a list of detected objects rather than as a full COCO dictionary. Each object contains:

- `segmentation`: run-length encoding (RLE) of a binary mask,
- `bbox`: bounding box $[x_{\min}, y_{\min}, w, h]$,
- `category_id`: class label,
- `score`: confidence score.

Unless otherwise specified, we use:

- crystals (grain body): `category_id = 1`,
- nuclei: `category_id = 2`,
- defects: `category_id = 3`.

2 Mask decoding, segmentation workflow, and per-grain morphology

For each object labeled as a crystal (category 1), the RLE is decoded into a binary mask $M(x, y)$ using `pycocotools`. The following geometry is extracted:

$$A = \sum_{x,y} \mathbf{1}_{M(x,y)}, \tag{1}$$

$$(c_x, c_y) = \frac{1}{A} \sum_{x,y} (x, y)\, \mathbf{1}_{M(x,y)}. \tag{2}$$

The perimeter P is estimated from a one-pixel morphological boundary, and the circularity is defined as

$$C = \frac{4\pi A}{P^2}. \tag{3}$$

The equivalent grain radius is defined throughout as

$$R_{\text{eq}} = \sqrt{\frac{A}{\pi}}. \tag{4}$$

Defect masks are accumulated into an image-level union mask $M_{\text{defect}}(x, y)$. The defect area within each grain is then

$$A_{\text{def}} = \sum_{x,y} \mathbf{1}_{M \wedge M_{\text{defect}}}, \tag{5}$$

yielding the defect fraction

$$\phi = \frac{A_{\text{def}}}{A}. \tag{6}$$

Here, and throughout, *defect* denotes optically defined intragrain regions (defect-like inclusions and secondary-contrast domains) identified from the per-grain heat-map representation, rather than directly resolved crystallographic point defects; the defect fraction ϕ is therefore an optical descriptor. For the texture-anisotropy analysis used in the main text, the intragrain optical texture is quantified from the local image-gradient field using the nematic ordering descriptor

$$A_{\text{tex}} = \frac{\left| \sum_{p \in \text{grain}} |\nabla I|_p e^{i2\theta_p} \right|}{\sum_{p \in \text{grain}} |\nabla I|_p}, \tag{7}$$

where $|\nabla I|_p$ and θ_p are the local image-gradient magnitude and orientation at pixel p, respectively. The factor $e^{i2\theta_p}$ enforces axial equivalence between θ_p and $\theta_p + \pi$, as appropriate for texture orientation. This is the texture-order quantity referred to as A_{tex} in the main-text descriptor table.

The whole-grain entropy reported in the main text is the heat-map texture entropy h_m, computed from the per-grain heat-map representation and expressed in bits per pixel. At the sample level h_m is statistically comparable between the two compositions (median 5.516 [5.506, 5.525] bits px^{-1} for FAPI and 5.525 [5.520, 5.530] for FAPI–TEMPO; Cliff's $\delta = -0.006$, two-sided Mann–Whitney $p = 0.77$). Two additional scalar entropy descriptors are retained in the exported dataset as companions: *entropy(bits)*, the Shannon entropy of the intensity distribution within the grain support, and *entropy_norm(bits)*, its normalized counterpart, which preserves the same grain-level ordering as *entropy(bits)* and therefore serves as a robustness companion rather than a conceptually independent observable. These intensity-based companions are higher in FAPI–TEMPO (Tables SI1 and SI2); because they summarize the raw intensity distribution rather than heat-map texture, this reflects a difference in observable rather than a contradiction with h_m. All three are whole-grain scalar summaries. By contrast, the annular entropy analysis used in the main text resolves the radial distribution of texture entropy within each grain, reported in raw bits per pixel, and is therefore locally sensitive to where disorder is concentrated.

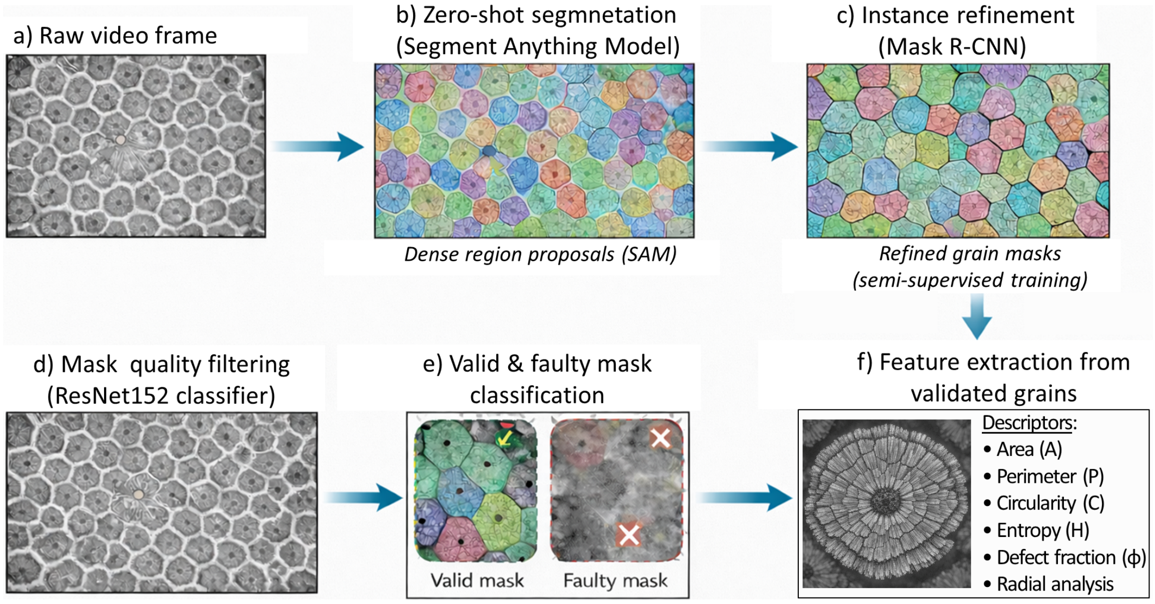

Figure SI1: **Segmentation-informed workflow for extracting grain-resolved microstructural descriptors from bright-field microscopy of FIRA-crystallized perovskite films.** (a) Representative bright-field frame from a crystallization video showing densely packed spherulitic grains in a FAPI-based perovskite film. (b) Dense region proposals generated by the Segment Anything Model (SAM) provide initial candidate masks for individual grains across the field of view. (c) A semi-supervised Mask R-CNN model in the Detectron2 framework refines these proposals into high-quality instance masks corresponding to individual spherulites. (d) A ResNet152-based mask-quality classifier filters the predicted masks to remove faulty segmentations, such as incomplete grains, overlapping regions, and ambiguous boundaries, ensuring that only physically consistent crystal domains are retained. (e) From each validated grain mask we extract geometric and structural descriptors including area A, perimeter P, circularity C, heat-map texture entropy h_m, and defect fraction ϕ. The grain area is also converted to an equivalent circular radius R_{eq}, enabling radial sampling using the normalized coordinate r/R_{eq}. These descriptors form the basis for the reconstruction of crystallization kinetics and the spatial fingerprint analyses presented in the main text.

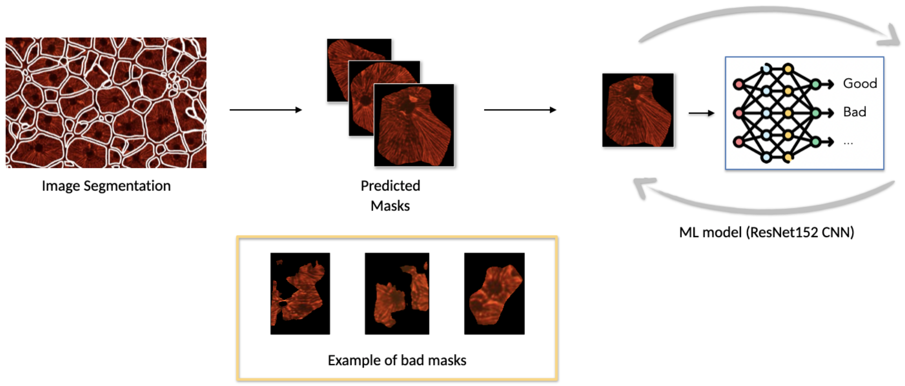

Figure SI2: **Mask-quality classifier examples used for post-segmentation validation.** Representative crops used to train and validate the ResNet152-based classifier that separates physically meaningful grain masks from faulty segmentations. (a) Examples of valid masks, corresponding to isolated grains with closed boundaries, internally consistent textures, and unambiguous grain assignment. (b) Examples of faulty masks, including incomplete domains, touching-edge grains, overlapping objects, ambiguous boundaries, and segmentation artefacts. This classifier constitutes the final quality-control stage before quantitative descriptor extraction.

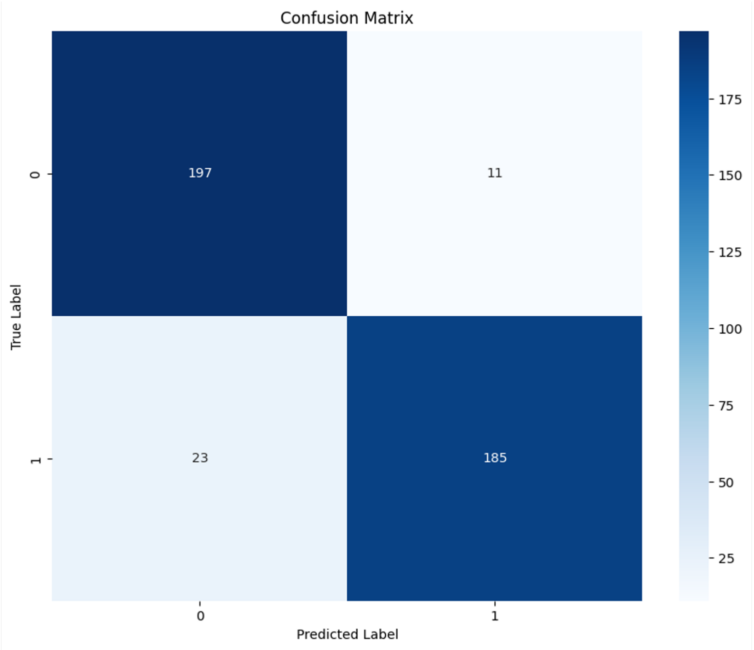

Figure SI3: **Performance of the mask-quality classifier on a held-out test set.** Confusion matrix for the binary ResNet152-based classifier distinguishing valid and faulty grain masks. (a) Faulty masks correctly identified as faulty. (b) Faulty masks misclassified as valid. (c) Valid masks misclassified as faulty. (d) Valid masks correctly identified as valid. The strong concentration along the diagonal indicates high precision and recall for both classes, confirming that the classifier reliably filters erroneous masks before downstream kinetic and structural analyses.

3 Grain-scale descriptor extensions

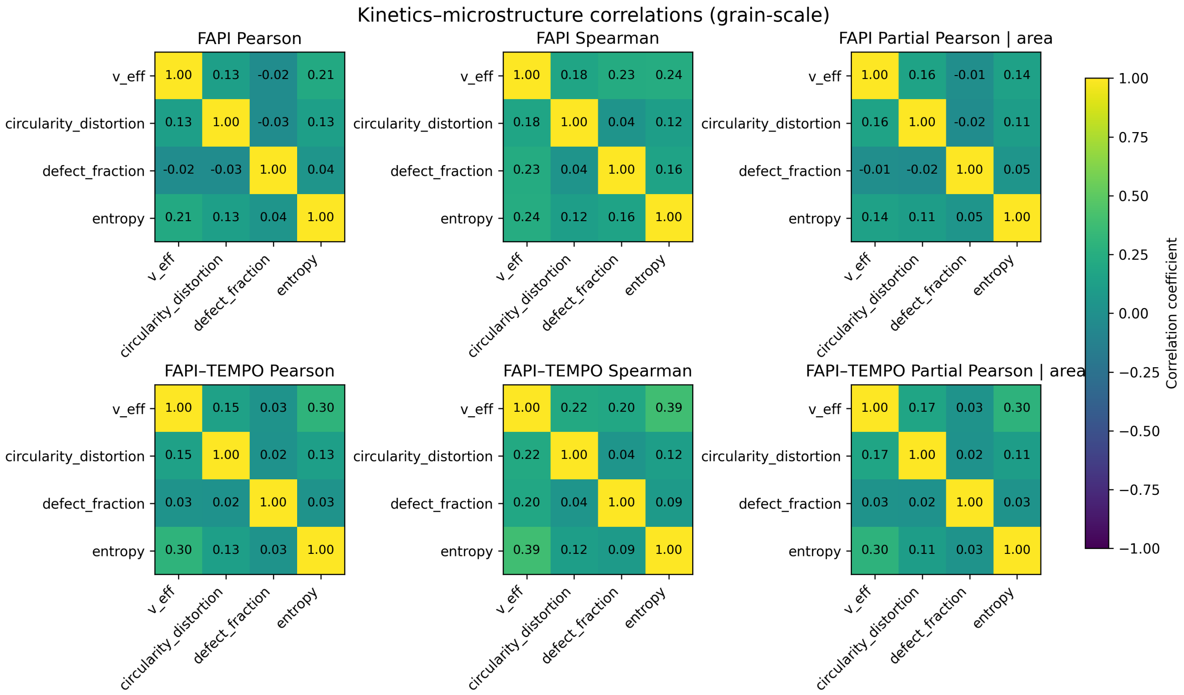

Figure SI4: **Grain-scale kinetics–microstructure correlation summary.** (a) Pearson correlation matrix for the variable set $\{v_R$, circularity distortion, ϕ, $H\}$ in FAPI. (b) Pearson correlation matrix for the same variable set in FAPI–TEMPO. (c) Spearman rank-based correlations capturing monotonic but non-linear associations across the full grain-level dataset. (d) Partial Pearson correlations after controlling for grain area. Across both compositions, the coefficients remain small, indicating that effective growth rate is only weakly constrained by any single static descriptor and that circularity distortion, defect fraction, and texture entropy h_m encode largely distinct aspects of the non-equilibrium growth response.

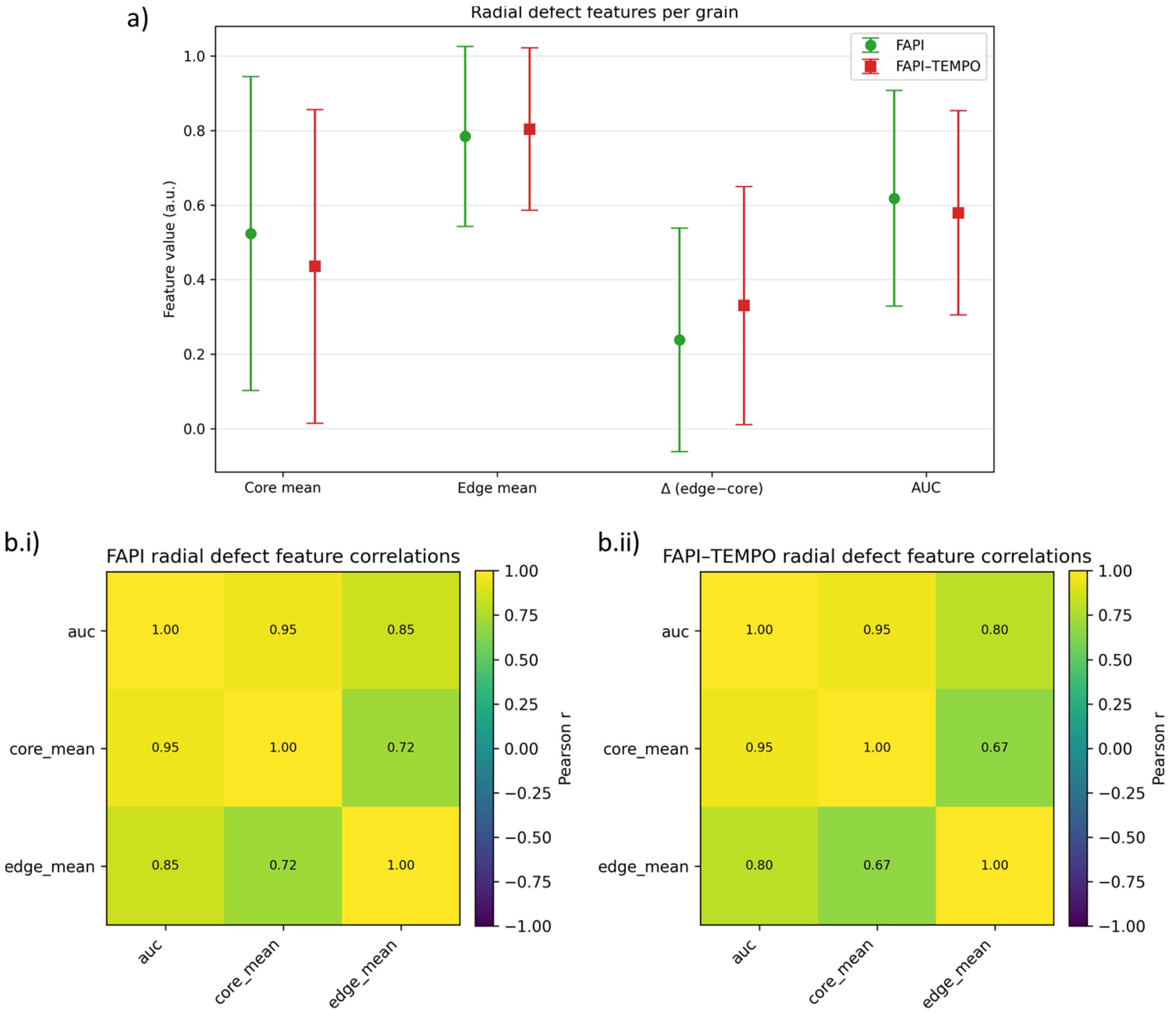

Figure SI5: **Scalar summaries of radial defect profiles and their internal coupling.** (a) Comparison of core mean, edge mean, Δ (edge minus core), and area under the curve (AUC) extracted from the per-grain radial defect profiles for FAPI and FAPI–TEMPO, shown as mean ± standard deviation. (b) Pearson correlation matrix among the scalar quantities {AUC, core mean, edge mean, Δ} for FAPI. (c) Corresponding Pearson correlation matrix for FAPI–TEMPO. Each per-grain radial defect-fraction profile $f(r/R_{\mathrm{eq}})$ is reduced to the scalar quantities $\langle f\rangle_{\mathrm{core}}$, $\langle f\rangle_{\mathrm{edge}}$, $\Delta = \langle f\rangle_{\mathrm{edge}} - \langle f\rangle_{\mathrm{core}}$, and $\mathrm{AUC} = \int_0^1 f(r/R_{\mathrm{eq}})\, d(r/R_{\mathrm{eq}})$. By construction, AUC correlates strongly with both core and edge means, while Δ remains the most discriminating compact indicator of front-localized defect accumulation.

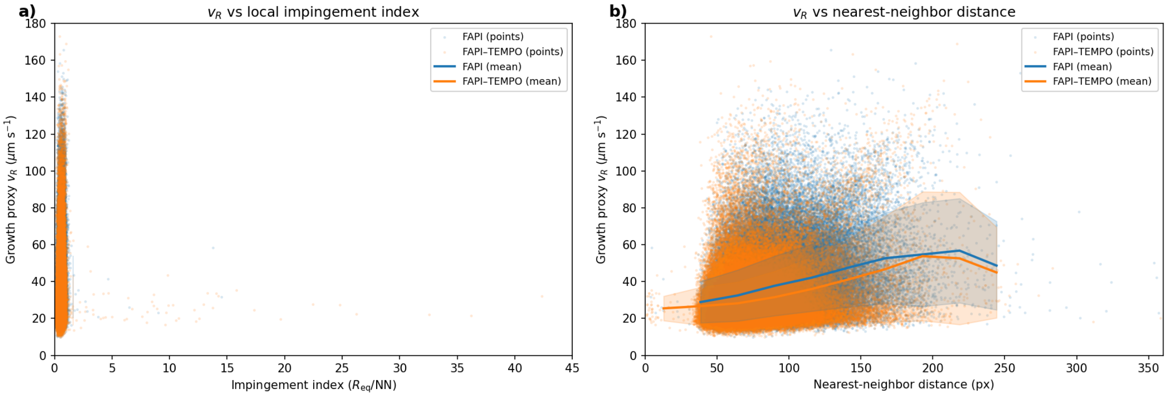

Figure SI6: **Grain-level crowding–kinetics associations.** (a) Growth proxy v_R as a function of the local impingement index $R_{\mathrm{eq}}/\mathrm{NN}$, where R_{eq} is the equivalent grain radius and NN is the nearest-neighbour distance between grain centroids. (b) Growth proxy v_R as a function of nearest-neighbour distance. Points correspond to individual grains from the FAPI and FAPI–TEMPO datasets, while solid curves denote the corresponding binned mean trends. The broad scatter indicates that local crowding geometry contributes to the kinetic landscape but does not uniquely determine the effective grain growth rate under flash infrared annealing.

4 Sample-level nonparametric statistics

4.1 Rationale

Because the full grain-level dataset contains many grains per micrograph and many micrographs per processed film, direct grain-level hypothesis testing would overstate the amount of independent statistical support. To address this, the principal between-composition comparisons were repeated using sample-level descriptor summaries, with each sample treated as an independent observational unit. This preserves the imaging-based character of the study while avoiding pseudoreplication at the individual-grain level.

For each sample and each descriptor, summary statistics were computed from the validated-grain population. The primary comparisons reported in the main text use the sample-level median for area, perimeter, equivalent radius, effective growth-rate proxy, the heat-map texture entropy h_m (with *entropy(bits)* and *entropy_norm(bits)* reported as companion descriptors), circularity distortion, and nucleus circularity. For defect fraction, the sample-level mean was used instead of the median because the distribution is strongly zero-inflated and the sample-level median is identically zero in both groups.

4.2 Statistical workflow

Between-group comparisons were carried out using two-sided Mann–Whitney U tests. Effect sizes were quantified with Cliff's delta, with positive values indicating larger values in FAPI and negative values indicating larger values in FAPI–TEMPO. Bootstrap 95% confidence intervals were calculated for the sample-level medians in each group and for the difference between group medians. Multiple-testing adjustment across the selected descriptor set was performed using the Benjamini–Hochberg false-discovery-rate procedure.

These sample-level results support the descriptive trends reported in the main text. Area, perimeter, equivalent radius, and effective growth-rate proxy are all larger in pristine FAPI, with large effect sizes. Circularity distortion, nucleus circularity, and defect-fraction mean are also higher in FAPI, but with smaller effect sizes. The primary whole-grain entropy descriptor, the heat-map texture entropy h_m, is statistically comparable between the two compositions

Table SI1: **Sample-level nonparametric comparison of the principal morphology and kinetics descriptors.** Medians are reported with bootstrap 95% confidence intervals. Positive Cliff's delta values indicate larger values in FAPI; negative values indicate larger values in FAPI–TEMPO.

Descriptor	FAPI	FAPI–TEMPO	Cliff's δ	Magnitude
Area median (μm^2)	1039.15 [1011.51, 1066.74]	682.28 [669.73, 694.42]	0.6211	large
Perimeter median (μm)	133.51 [131.77, 135.22]	107.58 [106.57, 108.72]	0.6139	large
Equivalent radius median (μm)	18.19 [17.94, 18.43]	14.74 [14.60, 14.87]	0.6211	large
v_{eff} median (μm s^{-1})	3.19 [3.15, 3.23]	2.59 [2.56, 2.61]	0.6601	large
Texture entropy h_m median (bits px^{-1})	5.516 [5.506, 5.525]	5.525 [5.520, 5.530]	-0.0062	negligible
Companion: entropy (intensity) median (bits)	5.7203 [5.7095, 5.7286]	6.2118 [6.2035, 6.2181]	-0.9424	large
Companion: normalized entropy median (bits)	5.7166 [5.7116, 5.7212]	6.2108 [6.2065, 6.2154]	-0.9963	large
Circularity-distortion median	0.2459 [0.2448, 0.2466]	0.2371 [0.2361, 0.2380]	0.2928	small
Nucleus-circularity median	0.3382 [0.3345, 0.3407]	0.3158 [0.3133, 0.3180]	0.2748	small
Defect-fraction mean	0.0048 [0.0045, 0.0050]	0.0026 [0.0024, 0.0028]	0.3084	small

Table SI2: **Sample-level median differences and significance.** Δ denotes FAPI minus FAPI–TEMPO.

Descriptor	Δ [95% CI]	p value	BH-FDR
Area median (μm^2)	356.87 [327.01, 386.93]	2.923×10^{-196}	6.100×10^{-196}
Perimeter median (μm)	25.93 [24.00, 27.91]	9.276×10^{-192}	1.855×10^{-191}
Equivalent radius median (μm)	3.45 [3.18, 3.73]	2.923×10^{-196}	6.100×10^{-196}
v_{eff} median (μm s^{-1})	0.60 [0.56, 0.65]	2.113×10^{-221}	1.691×10^{-220}
Texture entropy h_m median (bits px^{-1})	-0.008 [-0.020, 0.002]	0.77	0.77
Companion: entropy (intensity) median (bits)	-0.4915 [-0.5044, -0.4789]	$< 10^{-300}$	$< 10^{-300}$
Companion: normalized entropy median (bits)	-0.4942 [-0.5007, -0.4883]	$< 10^{-300}$	$< 10^{-300}$
Circularity-distortion median	0.0087 [0.0075, 0.0100]	4.459×10^{-45}	6.295×10^{-45}
Nucleus-circularity median	0.0224 [0.0178, 0.0261]	6.316×10^{-40}	8.662×10^{-40}
Defect-fraction mean	0.0022 [0.0019, 0.0025]	8.221×10^{-50}	1.233×10^{-49}

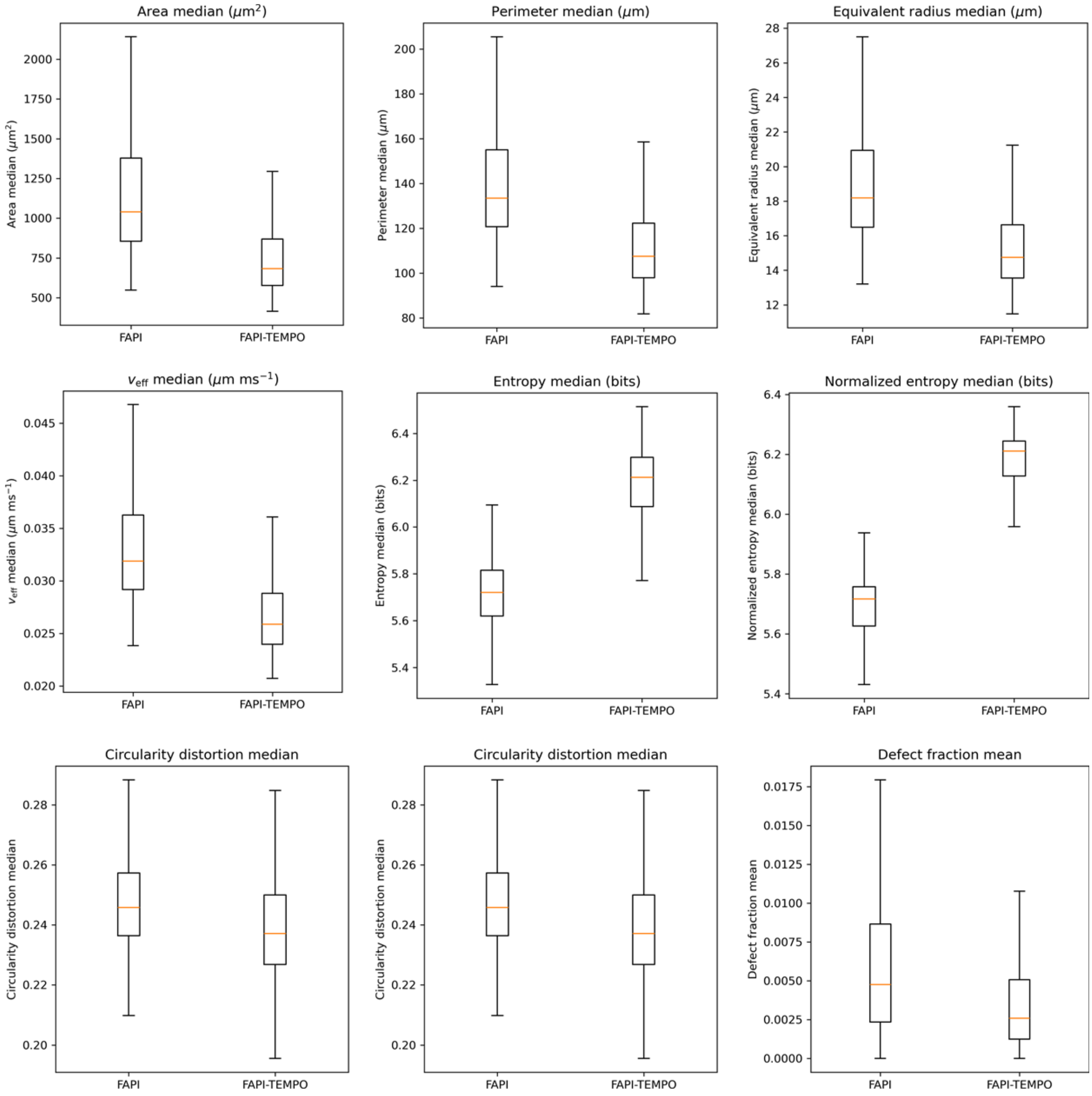

Figure SI7: **Sample-level distributions of the principal descriptors selected for non-parametric comparison.** Boxplots show the per-sample summary distributions for the descriptor set used in the final paper-level statistical comparison. Top row: area median, perimeter median, and equivalent radius median. Middle row: v_{eff} median and the two companion intensity-based scalar entropies (entropy median and normalized entropy median); the primary whole-grain descriptor h_m is statistically comparable between compositions and is reported in Table SI1. Bottom row: circularity-distortion median, nucleus-circularity median, and defect-fraction mean. These plots make clear that the between-composition contrasts are visible at the sample level rather than arising only from pooling very large grain populations.

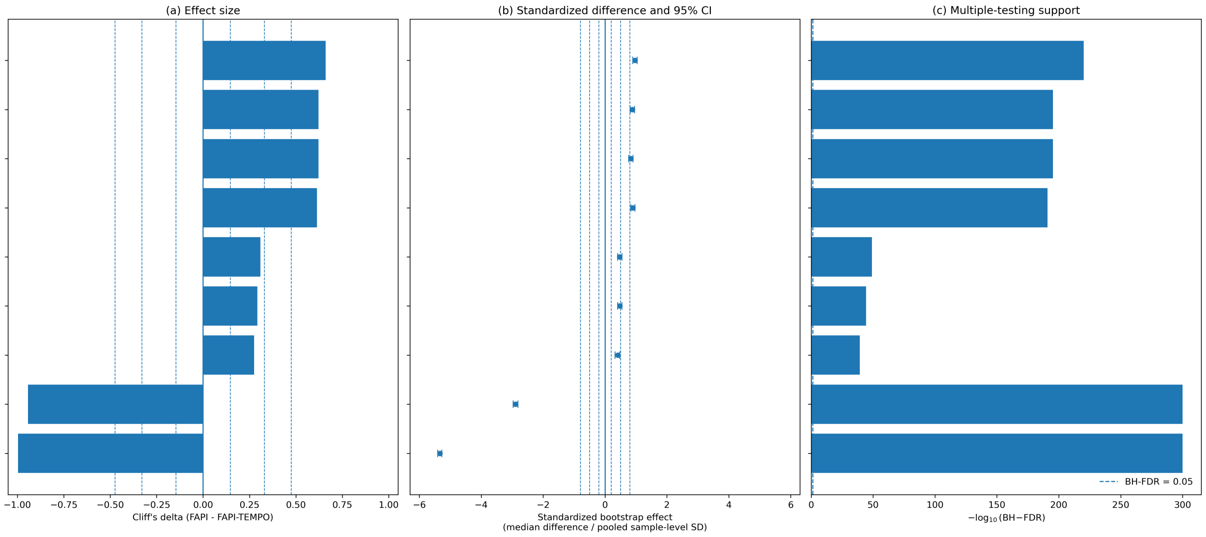

Figure SI8: **Integrated statistical summary of the principal sample-level descriptor comparisons.** (a) Cliff's delta effect sizes for FAPI minus FAPI–TEMPO. Positive values indicate larger values in FAPI and negative values indicate larger values in FAPI–TEMPO; the two negative entropy bars correspond to the intensity-based companion entropies, whereas the primary heat-map texture entropy h_m is statistically comparable between compositions ($\delta \approx 0$) and is reported in Tables SI1 and SI2. (b) Standardized bootstrap effect sizes, defined as the difference between group medians divided by the pooled sample-level standard deviation, together with bootstrap 95% confidence intervals. (c) Multiple-testing support summarized as $-\log_{10}$(BH-FDR) for the same descriptor set. Together, these panels separate effect magnitude, uncertainty, and adjusted significance for the sample-level nonparametric comparisons.

(Cliff's $\delta = -0.006$, $p = 0.77$). The two companion intensity-based scalar entropies are instead higher in FAPI–TEMPO with very large effect sizes; because they summarize the raw intensity distribution rather than heat-map texture, this reflects a difference in observable rather than a contradiction with h_m. Consistent with the main text, the annular decomposition of h_m shows that the inter-composition difference is concentrated in the inner and middle annuli—where pristine FAPI carries higher texture entropy—and converges at the outer growth front, with FAPI–TEMPO exhibiting the steeper inner-to-outer radial gradient. The whole-grain scalar and the radially resolved measures are therefore complementary rather than conflicting: a comparable whole-grain h_m and a composition-dependent radial profile describe different aspects of the same intragrain disorder.

5 Video-measured kinetics extraction

5.1 Video-derived observables

From time-resolved masks, we compute the event-count proxy $n(t)$, the event-rate proxy dn/dt, the transformed-fraction proxy $X(t)$ from segmented areas, and the transformation rate dX/dt. The operational definitions, including filtering rules, normalization choices, and smoothing settings, follow the implementation used to generate `counts_shifted.csv` and the burst-metric extraction workflow used in the main text. When temporal derivatives are required, the transformed-fraction or event-count traces are first smoothed using the same fixed preprocessing settings applied throughout the exported kinetics workflow, and only then differentiated. This avoids spurious oscillations from frame-to-frame segmentation noise while preserving the dominant burst structure. The underlying crystallization sequences are available as Supplementary Movie SM1 for pristine FAPI and Supplementary Movie SM2 for FAPI–TEMPO.

5.2 Representative pyrometer trace during FIRA

To provide direct information on the transient thermal environment during flash infrared annealing, we include a fast pyrometer trace acquired during FIRA processing. The measurement confirms that the film experiences a rapid thermal excursion with a well-defined maximum during the annealing event. The pyrometer was operationally calibrated against a direct contact reference: a fine-gauge (50 µm) K-type thermocouple with a digital readout, bonded to the FTO/glass substrate, recorded the substrate temperature under a 1 s calibration pulse comparable to and bracketing the 640 ms processing pulse. The emissivity was set to $\varepsilon = 0.90$—a physically reasonable value for the high-emissivity wet precursor film and forming α-$FAPbI_3$ absorber on the transparent conducting oxide—which gave agreement with the contact reference under this configuration, so the trace is interpreted as an approximate substrate-surface thermal history rather than an absolute film-temperature measurement. Because the pyrometer acquisition and the video-derived crystallization kinetics were not recorded under a rigorously synchronized timing protocol, owing to technical limitations associated with the ultrafast process, the trace is used to characterize the approximate transient thermal history and cooling behaviour rather than to extract a time-aligned crystallization-burst temperature.

5.3 Burst-metric extraction and quality control

To quantify the dominant crystallization burst from a single video per composition, we extract burst metrics including peak time, duration, full width at half maximum (FWHM), and synchrony descriptors from the video-derived $X(t)$ and $n(t)$ traces. For numerical stability, the shifted time axis is floored at 0 ms and the supporting quality-control diagnostics are reported explicitly below.

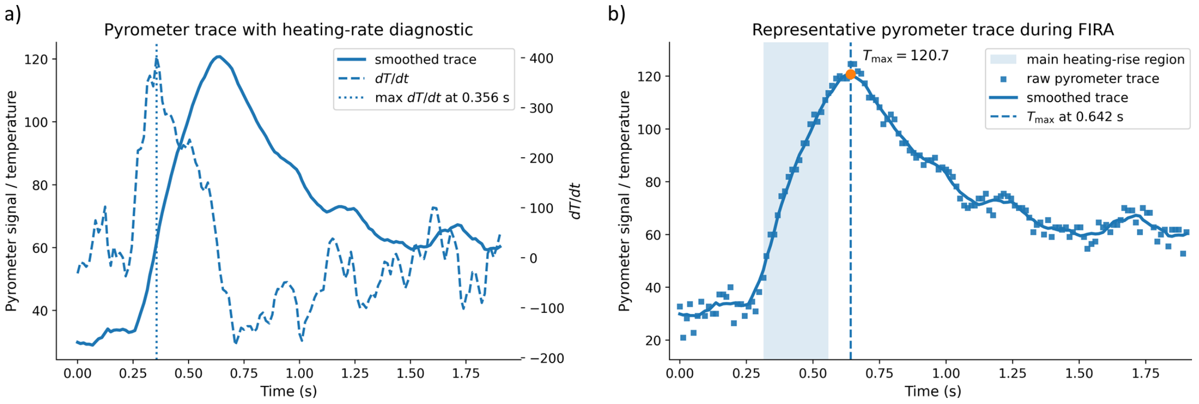

Figure SI9: **Representative pyrometer trace acquired during FIRA processing.** (a) Pyrometer trace with heating-rate diagnostic. The solid line shows the smoothed, operationally calibrated substrate-temperature profile, while the dashed line shows the corresponding derivative dT/dt. The vertical dotted line marks the time of maximum heating rate. (b) Representative pyrometer trace during FIRA. Square markers show the raw pyrometer data and the solid line shows the smoothed trace. The dashed vertical line marks the peak substrate temperature $T_{\max}$, and the shaded region indicates the main heating-rise interval.

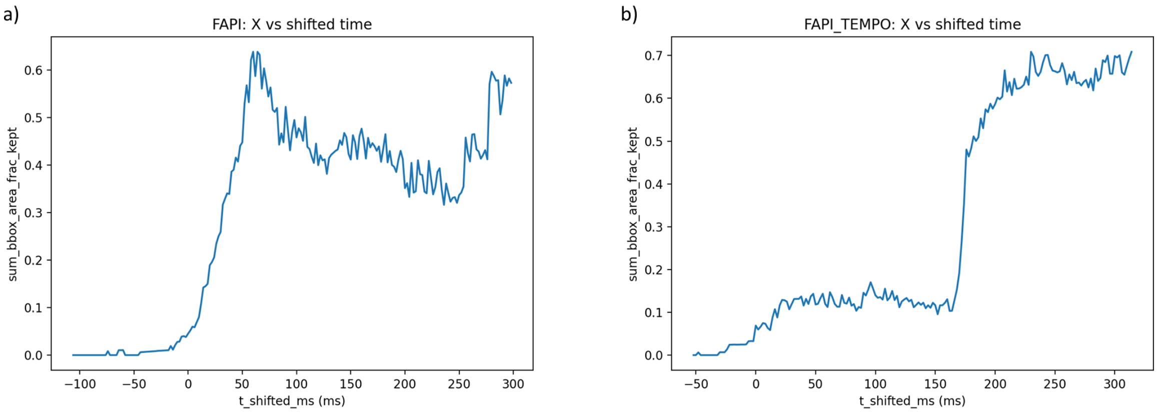

Figure SI10: **Quality-control diagnostics for the video-derived transformation fraction $X(t)$.** (a) Video-derived transformed fraction for FAPI after time shifting and normalization. (b) Corresponding transformed fraction for FAPI–TEMPO. (c) Diagnostic comparison between raw and monotonic-envelope representations used to assess whether cumulative transformation remains physically consistent. (d) Final normalized $X(t)$ traces used as the basis for subsequent burst extraction and comparative kinetics analysis.

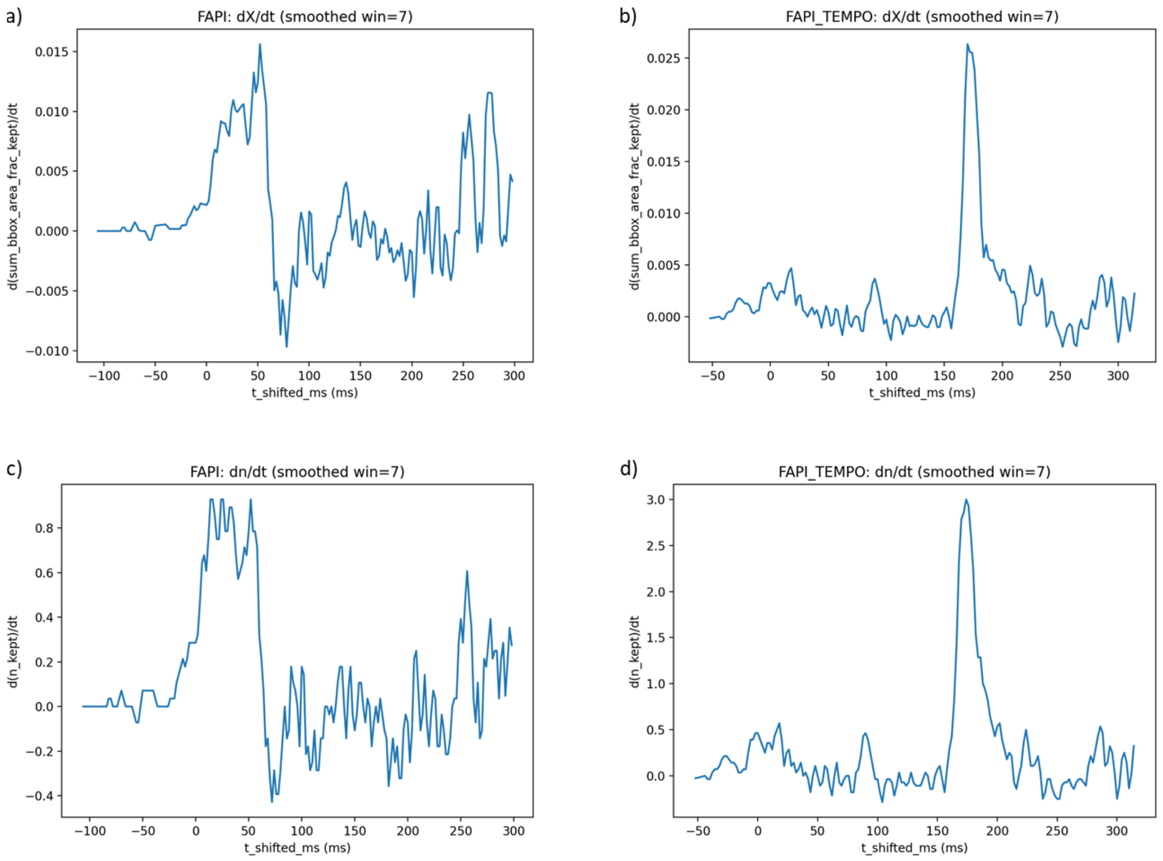

Figure SI11: **Video-derived rate curves and burst annotations.** (a) Transformation-rate curve dX/dt for FAPI, highlighting the dominant crystallization burst. (b) Transformation-rate curve dX/dt for FAPI–TEMPO. (c) Event-rate proxy dn/dt used to localize the dominant burst window and nucleation activity. (d) Annotated burst windows and peak markers used to extract peak time, width, and synchrony descriptors from the video-derived kinetics.

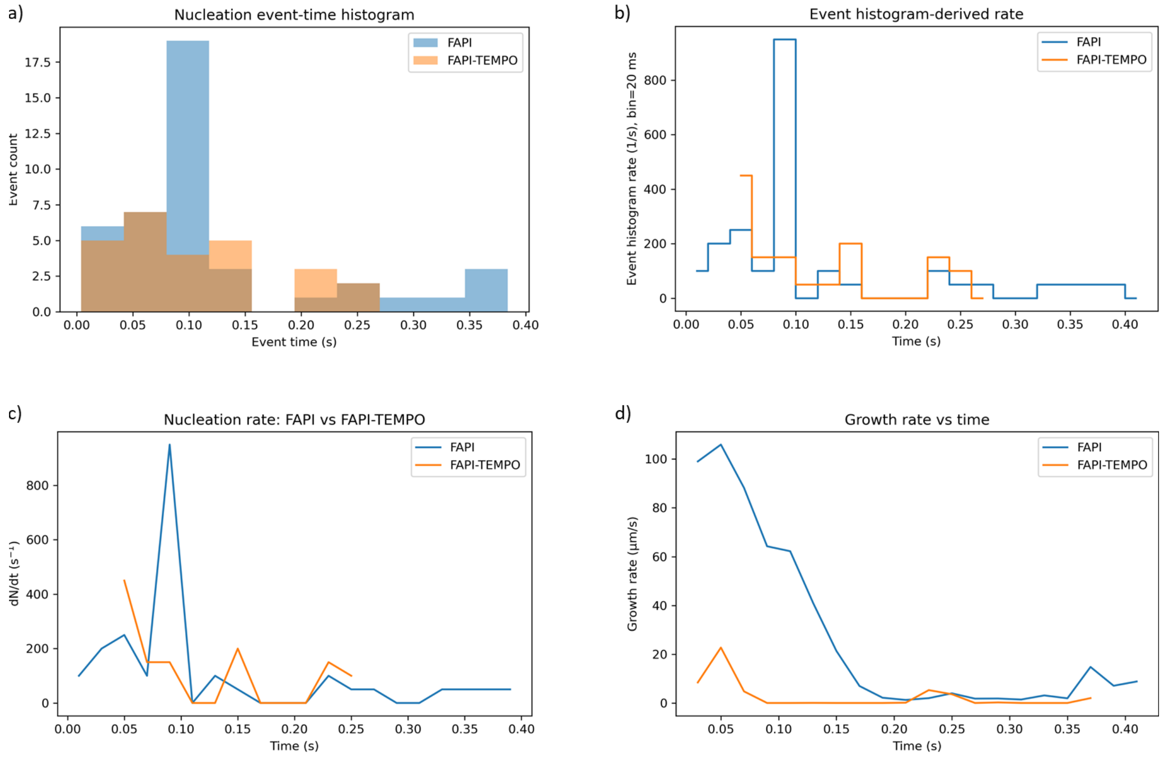

Figure SI12: **Event-time histograms supporting the burst interpretation of video-derived kinetics.** (a) Histogram of detected event times for FAPI. (b) Histogram of detected event times for FAPI–TEMPO. (c) Histogram-derived event-rate estimate for FAPI. (d) Histogram-derived event-rate estimate for FAPI–TEMPO. These plots provide a complementary discrete-event view of the same burst-like kinetics summarized in the continuous $X(t)$ and dX/dt representations.

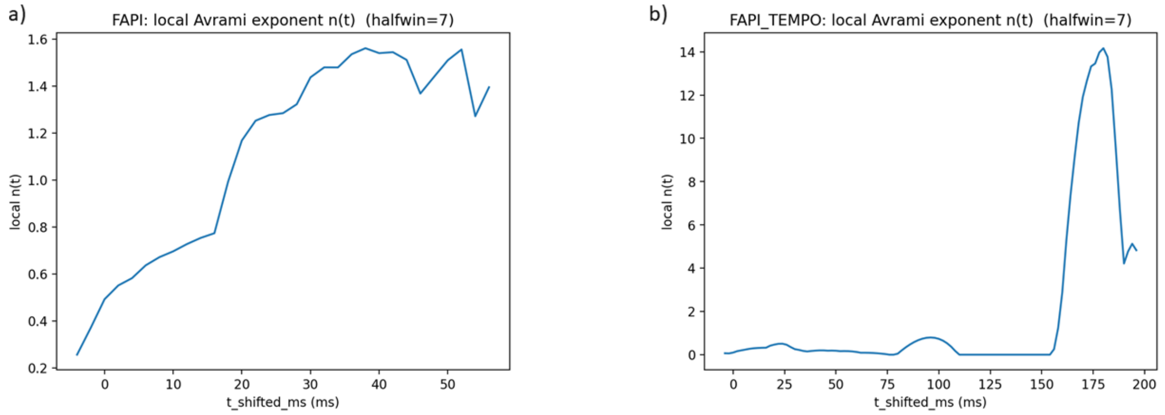

Figure SI13: **Local Avrami exponent reconstructed from the video-derived transformation curves.** (a) Time-dependent local Avrami exponent $n(t)$ calculated for FAPI from the video-derived transformation fraction. (b) Corresponding local Avrami exponent $n(t)$ for FAPI–TEMPO. The exponent is estimated from the differential Avrami relation $n(t) = \frac{d \ln[-\ln(1-X)]}{d \ln t}$ using a sliding-window procedure. The strong temporal variations in $n(t)$ reflect the burst-like, non-ideal, and non-isothermal character of FIRA crystallization.

6 Morphology-transported kinetics bridge

6.1 Rationale and hierarchy of statistical support

Because only one kinetics video exists per composition, statistical support for kinetics-like descriptors is obtained from the large static datasets using the hierarchy

$$\text{Grain} \rightarrow \text{ROI} \rightarrow \text{Micrograph} \rightarrow \text{Sample}.$$

The morphology-transported kinetics bridge maps static morphology distributions to an effective time axis anchored by the single-video kinetics, enabling reconstructed curves $n(t)$, dn/dt, $X(t)$, and dX/dt with uncertainty bands derived from ROI- or micrograph-level resampling.

6.2 Transport definition

In the transported representation, grain area or equivalent radius is used as the state variable. A monotone mapping between state and time is anchored to the video-derived kinetics so that static morphology distributions can be expressed as time-parametrized kinetics-like curves. Different weighting schemes, including count-weighted, area-weighted, and optional R^2-weighted variants, are evaluated for robustness.

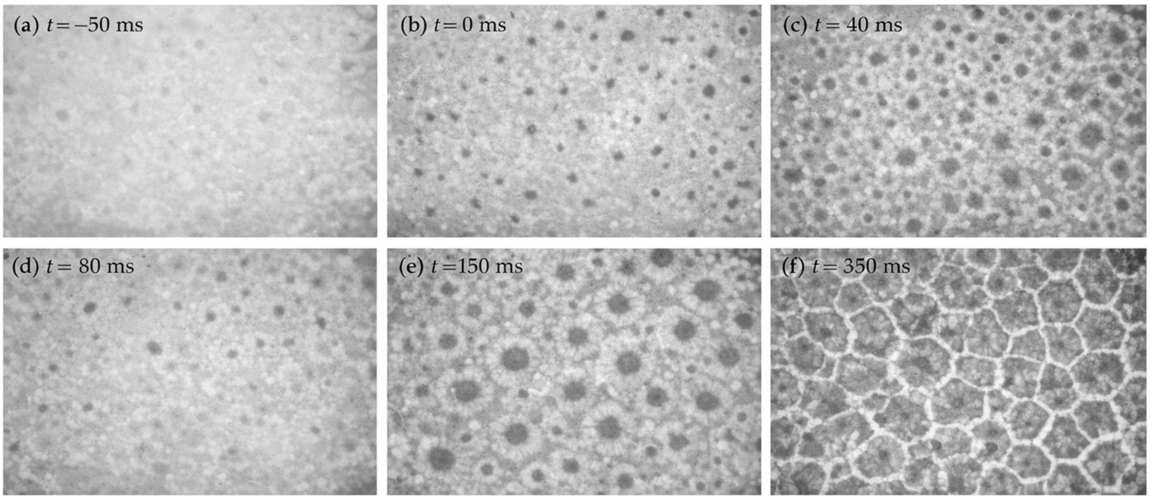

Figure SI14: **Schematic of the morphology-transported kinetics bridge.** (a) Video-derived anchor curves define the experimental crystallization timeline. (b) Static grain morphology distributions define the transported state variable. (c) A monotonic mapping is constructed between morphology state and effective time. (d) Static morphology distributions are transported onto the effective time axis to reconstruct $X(t)$ and $dX/dt(t)$ with uncertainty inherited from ROI- and micrograph-level variability.

6.3 Weighting robustness

We compare transported curves reconstructed under count-weighted, area-weighted, and optional R^2-weighted schemes. The main text uses the area-weighted representation as the primary descriptor; alternative weightings and their influence on the transported kinetics are reported here.

6.4 Transported Avrami fits

Shifted Avrami fits are applied to the transported curves as compact effective descriptors. Because the underlying FIRA process is non-isothermal and burst-like, the resulting parameters are

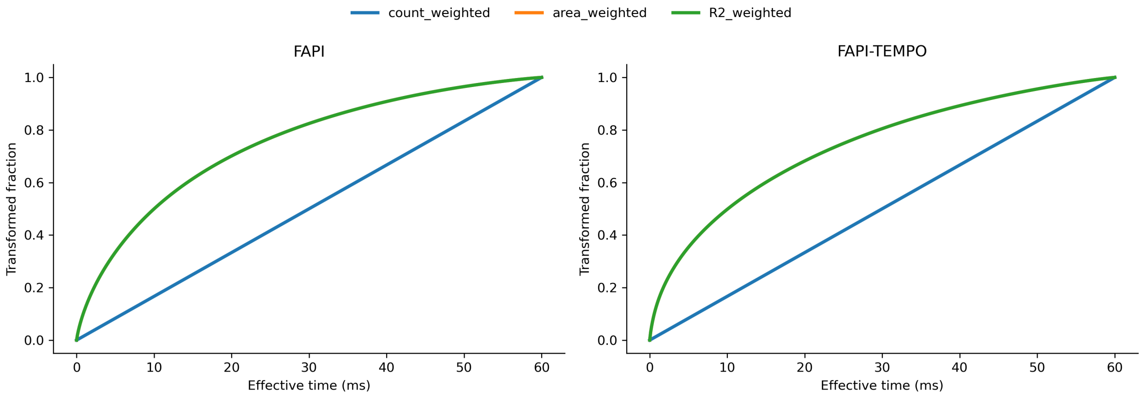

Figure SI15: **Weighting robustness of transported transformation curves.** (a) Morphology-transported transformation fraction $X(t)$ for FAPI reconstructed using count-weighted, area-weighted, and R^2-weighted schemes. (b) Corresponding transported $X(t)$ comparison for FAPI–TEMPO. The close agreement between the area-weighted and R^2-weighted curves confirms that the transported transformation profiles are robust to weighting choice, while the area-weighted representation is retained in the main text for physical interpretability.

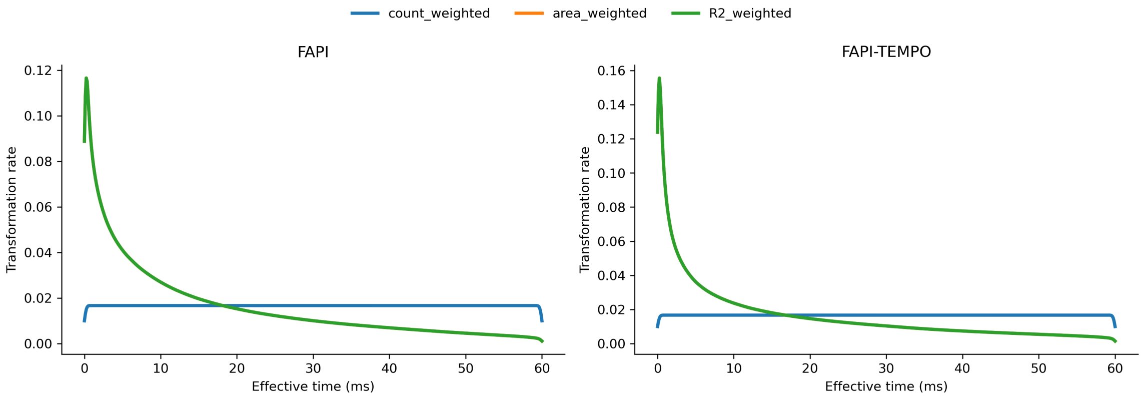

Figure SI16: **Weighting robustness of transported transformation-rate curves.** (a) Morphology-transported transformation rate $dX/dt(t)$ for FAPI reconstructed using count-weighted, area-weighted, and R^2-weighted schemes. (b) Corresponding transported transformation-rate comparison for FAPI–TEMPO. The similar shapes and relative peak positions across weighting schemes show that the main conclusions do not depend on the specific weighting definition used in the transport step.

interpreted comparatively between compositions rather than mechanistically as literal nucleation dimensionalities.

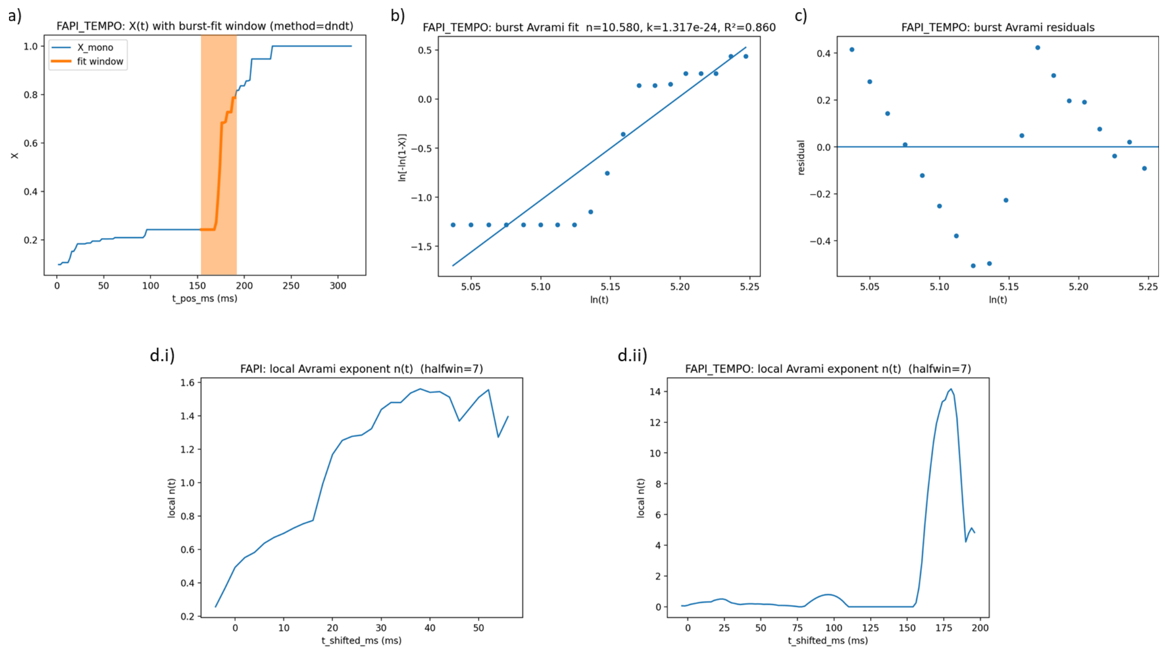

Figure SI17: **Effective Avrami descriptors of the morphology-transported kinetics.** (a) Morphology-transported transformation curve $X(t)$ together with the effective Avrami fit used to summarize the FAPI kinetics. (b) Corresponding effective Avrami fit for the FAPI–TEMPO transported transformation curve. (c) Linearized effective Avrami representation used to estimate the apparent exponent and rate constant over the selected fitting window. (d) Residuals of the effective Avrami fit, showing the degree to which the transported kinetics deviate from an ideal sigmoidal Avrami form. These fits are used as compact effective descriptors of the transported kinetics only; because FIRA crystallization is burst-like and non-isothermal, the extracted parameters are not interpreted as strict mechanistic exponents.

7 Spatial fingerprints and descriptor-level statistics

7.1 Canonical grain construction and radial averaging

This section describes how per-grain descriptor fields are mapped onto a normalized radial coordinate to compare internal and outer-front regions across grains of different size. For each validated grain, intragrain quantities are mapped onto the normalized radius r/R_{eq}, with R_{eq} the equivalent grain radius, and averaged within annuli before averaging over the ensemble. Unless otherwise specified, scalar descriptor values are reported as mean $\pm$ standard deviation across grains, radial solid lines denote the average across grains at each normalized radius, and the shaded bands in radial plots represent the standard deviation across grains at each radial bin.

Nearest-neighbour distance (NN) is defined as the centroid-to-centroid distance between a given grain and its closest neighbouring grain within the same field of view. The corresponding impingement proxy is expressed as $R_{\mathrm{eq}}/\mathrm{NN}$, so larger values indicate a more crowded local growth environment.

7.2 Updated radial kinetic heterogeneity proxy

The main text uses an annulus-conditioned radial kinetic heterogeneity proxy, computed as $\mathrm{CV}(v_R)$ conditioned on r/R_{eq}. This quantity should be interpreted as a spatially correlated, annulus-conditioned grain-level proxy for growth-rate dispersion rather than as a pixelwise local transformation-rate field. The corresponding SI quality-control figure verifies that the radial bins remain well supported across the grain radius.

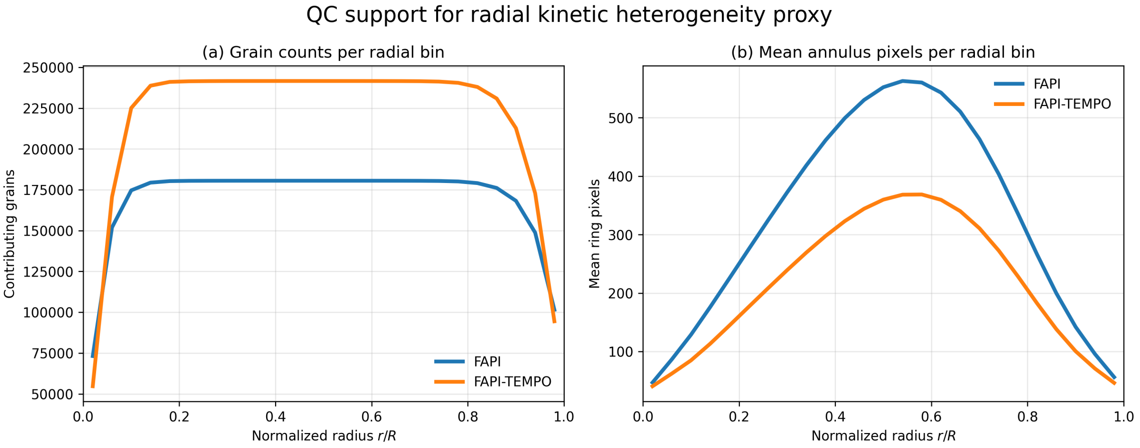

Figure SI18: **Quality-control support for the radial kinetic-heterogeneity proxy.** (a) Number of grains contributing to each normalized radial bin r/R_{eq} used to compute the annulus-conditioned $\mathrm{CV}(v_R)$ statistics. (b) Mean number of contributing pixels per radial annulus as a function of r/R_{eq}. These diagnostics confirm that the radial proxy reported in the main text is supported by sufficient grain counts and spatial sampling across the full normalized radius for both FAPI and FAPI–TEMPO.

7.3 Caption-ready quantification of radial curves

To convert radial fingerprints into compact comparative descriptors, each radial curve is quantified by its centre-to-mid change, mid-to-edge change, edge-minus-centre contrast, and area under the curve (AUC). The exported SI tables provide both per-sample metrics and pairwise contrasts defined as FAPI minus FAPI–TEMPO.

Table SI3: Caption-ready quantification metrics for radial curves, reported separately for each sample.

Curve	Sample	centre→mid	mid→edge	edge−centre	AUC
NN distance (μm)	FAPI	-0.690	0.753	0.062	36.400
NN distance (μm)	FAPI–TEMPO	-0.616	0.537	-0.079	30.060
NN distance (px)	FAPI	-1.519	1.656	0.137	80.086
NN distance (px)	FAPI–TEMPO	-1.355	1.182	-0.173	66.137
Impingement index (R_{eq}/NN)	FAPI	-0.0072	0.0090	0.0018	0.5461
Impingement index (R_{eq}/NN)	FAPI–TEMPO	-0.0086	0.0064	-0.0022	0.5030
Median v_R (μm s^{-1})	FAPI	-2.83	3.15	0.32	31.06
Median v_R (μm s^{-1})	FAPI–TEMPO	-3.71	3.49	-0.22	25.82
Kinetic heterogeneity CV(v_R)	FAPI	0.04325	-0.03732	0.00593	0.43105
Kinetic heterogeneity CV(v_R)	FAPI–TEMPO	0.04419	-0.02674	0.01745	0.42151

Table SI4: Pairwise radial-curve contrasts defined as FAPI minus FAPI–TEMPO.

Curve	Δ(centre→mid)	Δ(mid→edge)	Δ(edge−centre)	ΔAUC
NN distance (μm)	-0.074	0.215	0.141	6.340
NN distance (px)	-0.163	0.473	0.310	13.949
Impingement index (R_{eq}/NN)	0.0014	0.0026	0.0040	0.0431
Median v_R (μm s^{-1})	0.88	-0.34	0.54	5.24
Kinetic heterogeneity CV(v_R)	-0.00094	-0.01058	-0.01153	0.00954

8 Literature benchmarking and scope of inferred mechanisms

8.1 Positioning of the present descriptor framework

Recent advanced studies of halide-perovskite crystallization have clarified important but often separate aspects of process physics. Some works resolve continuous phase-conversion trajectories directly by in situ structural and optical monitoring; others focus on rapid annealing or FIRA-like growth, on process-physics competition between drying and crystallization, or on additive-mediated coarsening and secondary grain growth. The present workflow is positioned relative to that literature as a descriptor-rich, segmentation-informed bridge between direct video kinetics and statistically resolved final-state microstructure.

The key contribution of the present dataset is that several classes of observables are extracted within the same experimental system: video-derived transformed-fraction and rate curves, burst metrics, grain-level effective growth-rate distributions, morphology-conditioned growth trends, radial defect and entropy fingerprints, crowding descriptors, and morphology-transported effective kinetic summaries. This gives broader structural coverage than a single scalar fit, but narrower mechanistic specificity than a coupled thermofluidic or phase-field model.

Accordingly, the present framework supports physically motivated interpretation of delayed dominant bursts, narrowed growth-rate landscapes, front-localized defect accumulation, and crowding-conditioned growth constraints. By contrast, it does not on its own constitute a direct measurement of quantities such as the evaporation-to-crystallization rate ratio, an absolute heterogeneous-nucleation barrier, or a full coarsening law. Those require either independent solvent/thermal observables or a more explicit forward model of film formation.

8.2 Mechanistic effects that may be constrained indirectly

Within these limits, several mechanistic scenarios can still be probed indirectly from the present dataset:

- **Heterogeneous nucleation bias.** A shift in burst timing, early event structure, and radial front statistics can indicate modified nucleation-site activation under additive treatment, although the dataset does not independently localize all heterogeneous nucleation sources.
- **Secondary nucleation or multi-stage activation.** A broadened or multi-feature event-rate structure, or a decoupling between early transformed-fraction increase and later growth-rate maxima, can be consistent with secondary activation processes.
- **Late-stage coarsening or grain-growth continuation.** Morphology distributions and shape evolution may reveal whether growth continues after the main transformation burst, but establishing true Ostwald ripening requires explicit evidence of small-domain shrinkage feeding large-domain growth.
- **Crowding-limited front propagation.** The combined use of nearest-neighbour distance, impingement index, and annulus-conditioned v_R provides a practical measure of how local geometric confinement narrows the accessible growth landscape.
- **Drying-versus-crystallization competition.** The present dataset is well suited to motivate that competition as an organizing principle, but a true ratio between evaporation and crystallization rates would require independent calibration of solvent-loss kinetics, thickness evolution, or thermal history.

8.3 Benchmarking table

Table SI5 summarizes how the present workflow compares with representative advanced directions in the recent literature. The purpose is not to claim direct equivalence between observables measured in different systems, but to show where the present framework contributes a useful and relatively uncommon combination of kinetic, morphological, and spatially resolved descriptors.

8.4 On the evaporation-to-crystallization rate ratio

Recent process-physics work has argued that the ratio between evaporation and crystallization rates can act as a governing control parameter for final morphology. This is an attractive concept for interpreting the present results, because the observed contrast between pristine FAPI and FAPI–TEMPO includes both kinetic re-timing and final-state microstructural regularization. However, within the current dataset this ratio cannot yet be extracted as a primary measured quantity. Doing so would require at least one independently calibrated observable tied to solvent removal or thickness evolution, for example:

- in situ thickness evolution,
- mass-loss or solvent-loss tracking,
- calibrated thermal transients under the FIRA pulse,
- an optical proxy demonstrably proportional to liquid-to-solid conversion rather than only segmented grain area.

If such observables are added in future work, the present descriptor framework would be well suited to connect that ratio to the resulting growth-rate landscape, radial disorder localization, and front-shape fingerprints. For the current study, the safer interpretation is that the results are consistent with altered competition among drying, nucleation, growth, coarsening, and impingement timescales, rather than constituting a direct measurement of their ratio.

Table SI5: **Benchmarking perspective for the present framework relative to advanced crystallization literature.** The final column indicates whether the corresponding quantity is directly measured here, indirectly constrained, or outside the scope of the present dataset.

**Literature direction**	**Representative study**	**Representative emphasis**	**What the present work adds or complements**	**Status here**
Continuous in situ crystallization trajectory studies	Hu *et al.*, *Nat. Commun.* **8**, 15688 (2017), DOI: https://doi.org/10.1038/ncomms15688	Direct real-time observation of perovskite crystallization from precursor solution to final film, linking phase evolution, morphology evolution, and depletion-driven pattern formation.	Adds grain-resolved segmentation, burst descriptors, morphology-conditioned growth statistics, and connection to a much larger static image population.	Directly measured / extended
Rapid thermal or FIRA-like in situ crystallization studies	Günzler *et al.*, *ACS Appl. Mater. Interfaces* **13**, 15518–15527 (2021), DOI: https://doi.org/10.1021/acsami.0c20958	In situ crystallization mechanism of rapidly thermally annealed prepatterned perovskite films, emphasizing nucleation control, rapid domain development, and microstructure formation under strong driving.	Adds morphology transport, radial fingerprints, descriptor-conditioned growth analysis, and local crowding metrics in a FIRA-relevant kinetic framework.	Directly measured / extended
Process-physics studies of drying vs crystallization	Majewski *et al.*, *Mater. Horiz.* **12**, 555–564 (2025), DOI: https://doi.org/10.1039/D4MH00957F	Coupled in situ / ex situ characterization and phase-field-based process simulation; identifies the ratio of evaporation to crystallization rates as a key morphology-control parameter.	Provides kinetic and microstructural descriptors that can later be compared to such models, but does not directly measure solvent-loss rate, film-thickness evolution, or the evaporation-to-crystallization ratio itself.	Indirectly relevant only
Additive-mediated coarsening or secondary growth studies	Maschwitz *et al.*, *Nat. Commun.* **16**, 9894 (2025), DOI: https://doi.org/10.1038/s41467-025-65484-7	Shows that common crystallization additives may not predominantly alter nucleation, but instead facilitate coarsening grain growth by increasing ion mobility across grain boundaries.	Provides a descriptor language to detect delayed bursts, narrowed growth landscapes, altered final-state distributions, and radial front signatures consistent with post-nucleation restructuring.	Indirectly inferred
Classical JMAK / Avrami parameterization studies	Fanfoni and Tomellini, *Il Nuovo Cimento D* **20**, 1171–1182 (1998), DOI: https://doi.org/10.1007/BF03185527; Shirzad *et al.*, *J. R. Soc. Interface* **20**, 20230242 (2023), DOI: https://doi.org/10.1098/rsif.2023.0242	Compact fitting and critical assessment of transformed-fraction curves through apparent exponents and rate parameters, together with explicit discussion of interpretive limits.	Uses Avrami-type fits as effective comparative descriptors while explicitly avoiding strict mechanistic over-interpretation under burst-like, non-isothermal FIRA conditions.	Directly reported as effective only
Polymer / solution-processing front-instability literature	Crist and Schultz, *Prog. Polym. Sci.* **56**, 1–63 (2016), DOI: https://doi.org/10.1016/j.progpolymsci.2015.11.006; Gránásy *et al.*, *Phys. Rev. E* **72**, 011605 (2005), DOI: https://doi.org/10.1103/PhysRevE.72.011605	Radial anisotropy, front roughness, branching, impingement, transport-limited morphology selection, and defect localization in spherulitic growth under strongly non-equilibrium conditions.	Imports this spatial-fingerprint perspective into halide perovskites using radial entropy, defect localization, texture anisotropy, polar anisotropy, and crowding-conditioned growth descriptors.	Directly extended

9 Representative true local pixelwise distance fields

To move beyond grain-resolved proxies and toward genuinely local spatial correlations, three true local pixelwise distance fields are constructed inside each grain using distance transforms: (i) distance-to-boundary, (ii) distance-to-defect, and (iii) distance-to-nucleus. Representative maps are included here to document the geometry encoded by these fields.

9.1 Local geometric distance fields

Beyond grain-averaged descriptors and radial fingerprints, the segmentation masks also allow the construction of fully local geometric fields inside each grain. These fields encode the spatial relationship between any intragrain pixel and key structural elements such as the grain boundary, defect domains, or the nucleation centre.

For a given grain mask $M(x, y)$, a Euclidean distance transform is applied to obtain spatial fields of the form

$$D_S(x, y) = \min_{(x',y') \in S} \sqrt{(x - x')^2 + (y - y')^2},$$

where S represents the reference set of pixels defining the structure of interest. In this work we compute three representative fields: the distance-to-boundary field ($S = \partial M$), the distance-to-defect field ($S = M_{\text{defect}}$), and the distance-to-nucleus field ($S = M_{\text{nucleus}}$).

These fields provide a geometrically complete description of the local environment within each crystal and constitute the basis for potential pixel-level analyses linking growth dynamics, defect localization, and optical texture. Representative examples of these distance maps are shown in Fig. SI19.

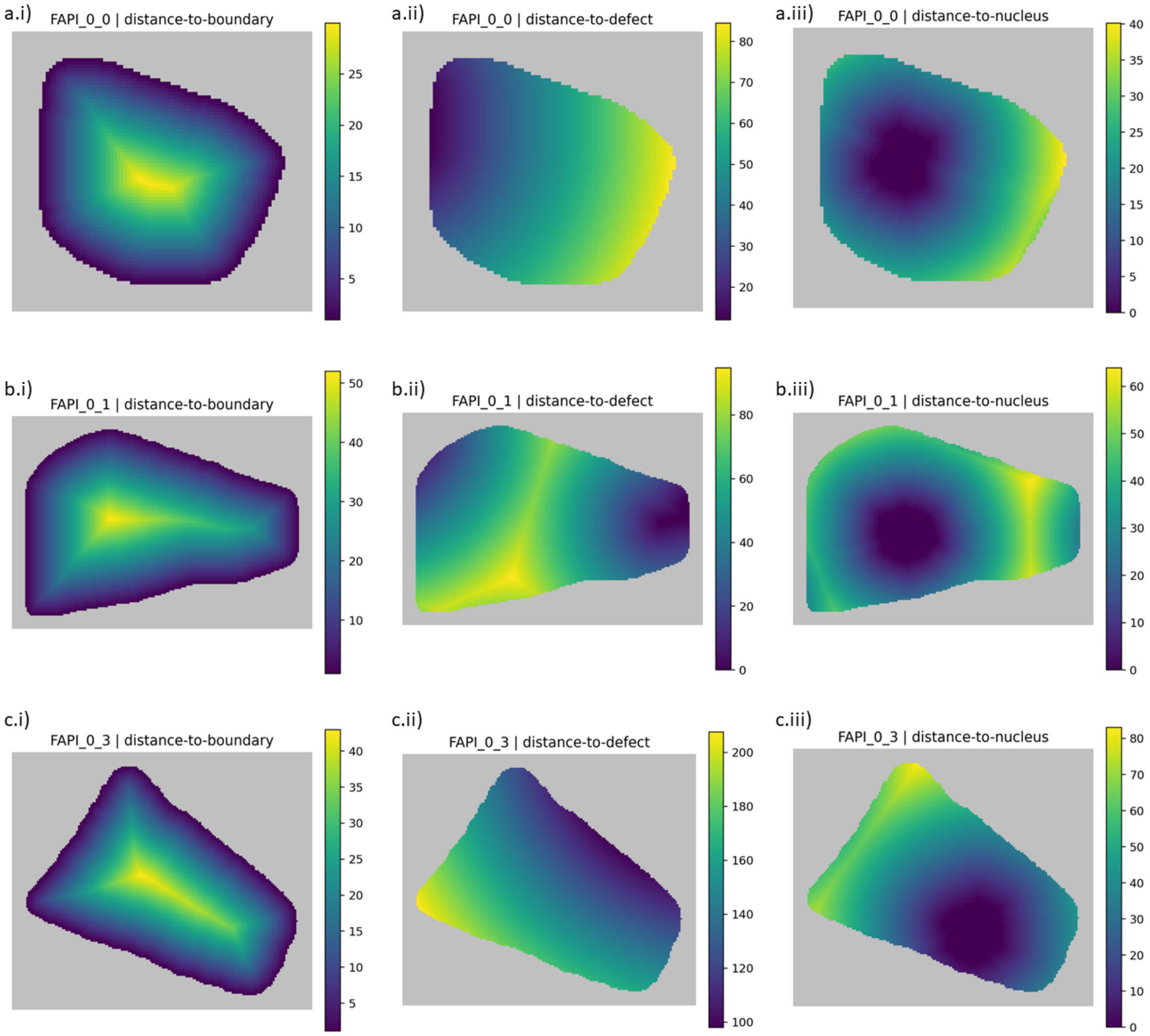

Figure SI19: **Representative local distance-field maps computed from segmented grain masks.** Rows show representative FAPI grains, while columns show the corresponding true local pixelwise distance fields computed from the same segmented objects: distance-to-boundary, distance-to-defect, and distance-to-nucleus. These maps illustrate how the local geometric environment is encoded directly from the grain, defect, and nucleus masks. The distance-to-boundary field captures the inward radial depth from the grain perimeter; the distance-to-defect field reports proximity to the nearest defect-containing region; and the distance-to-nucleus field resolves the spatial relationship between each intragrain pixel and the detected nucleation center. Together, these examples demonstrate that the local-field construction preserves spatially resolved geometric information within individual grains and provides the basis for future pixel-level structure–property analyses.

10 Outputs

The workflow exports:

- **Video-measured kinetics:** $X(t)$, $dX/dt(t)$, $n(t)$, $dn/dt(t)$, burst metrics, and quality-control plots, together with the corresponding raw crystallization sequences supplied as Supplementary Movies SM1 and SM2.
- **Transported kinetics:** reconstructed curves under different weightings, comparison metrics, bootstrap tables, effective Avrami descriptors, and compact kinetic summary quantities used in the main-text comparison table.
- **Radial fingerprints:** radial descriptor profiles, updated radial kinetic heterogeneity proxy with dispersion, grain-scale summary figures, crowding–kinetics association plots, and caption-ready radial summary tables.
- **Sample-level statistics:** Mann–Whitney U results, Cliff's delta effect sizes, bootstrap confidence intervals, compact paper-ready descriptor tables, and supporting plots.
- **Benchmarking support:** literature-positioning text and a benchmarking table clarifying which mechanistic quantities are directly measured, indirectly constrained, or outside the scope of the present dataset.
- **True local-field analyses:** representative distance-field maps constructed from grain, defect, and nucleus masks.

11 Structural and optical phase-validation figures

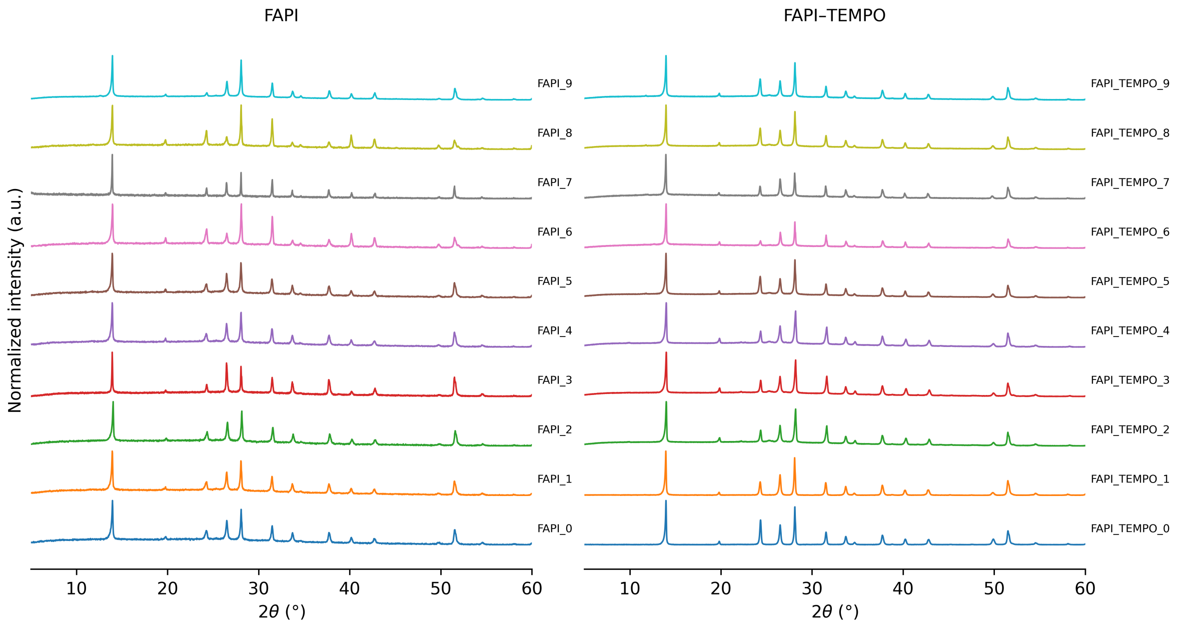

Figure SI20: **X-ray diffraction of fresh FIRA-crystallized FAPI and FAPI–TEMPO films.** Stacked XRD patterns for selected films showing α-$FAPbI_3$-dominant diffraction for both compositions. Patterns are normalized and vertically offset for clarity. The $\alpha(100)$ reflection near $2\theta \approx 14°$ is dominant and the higher-angle α-phase series is resolved, while residual δ-$FAPbI_3/PbI_2$ signatures remain minor.

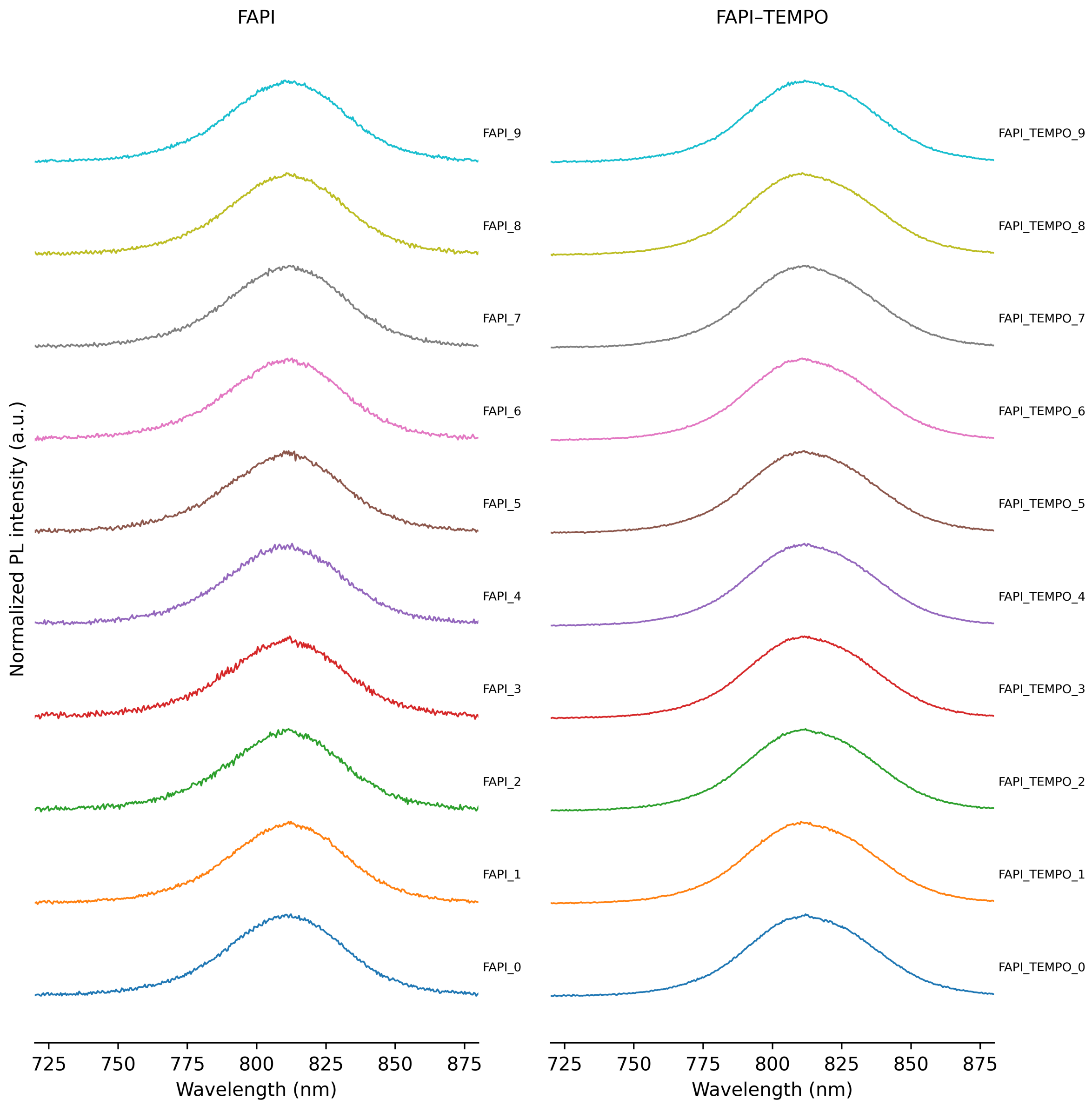

Figure SI21: **Steady-state photoluminescence of FAPI and FAPI–TEMPO films.** Stacked PL spectra for the corresponding FIRA-processed films. Spectra are baseline corrected, normalized, and vertically offset for clarity. Both compositions show reproducible near-infrared band-edge emission centred at approximately 811 nm, with no composition-dependent peak shift, supporting assignment to photoactive α-$FAPbI_3$.

References

[1] K. He, G. Gkioxari, P. Dollár, and R. Girshick, "Mask R-CNN," in *Proceedings of the IEEE International Conference on Computer Vision*, 2017, pp. 2980–2988.

[2] T.-Y. Lin *et al.*, "Microsoft COCO: Common Objects in Context," in *European Conference on Computer Vision*, 2014, pp. 740–755.

[3] A. N. Kolmogorov, "On the Statistical Theory of Crystallization of Metals," *Izvestiya Akademii Nauk SSSR, Seriya Matematicheskaya*, 1937.

[4] W. A. Johnson and R. F. Mehl, "Reaction Kinetics in Processes of Nucleation and Growth," *Transactions of the American Institute of Mining and Metallurgical Engineers*, vol. 135, pp. 416–442, 1939.

[5] M. Avrami, "Kinetics of Phase Change. I. General Theory," *The Journal of Chemical Physics*, vol. 7, no. 12, pp. 1103–1112, 1939.

[6] M. Avrami, "Kinetics of Phase Change. II. Transformation-Time Relations for Random Distribution of Nuclei," *The Journal of Chemical Physics*, vol. 8, no. 2, pp. 212–224, 1940.

[7] M. Avrami, "Granulation, Phase Change, and Microstructure. Kinetics of Phase Change. III," *The Journal of Chemical Physics*, vol. 9, no. 2, pp. 177–184, 1941.

[8] M. Fanfoni and M. Tomellini, "The Johnson–Mehl–Avrami–Kolmogorov model: a brief review," *Il Nuovo Cimento D*, vol. 20, pp. 1171–1182, 1998.

[9] K. Shirzad *et al.*, "A critical review on applications of the Avrami equation beyond materials science," *Journal of The Royal Society Interface*, vol. 20, article 20230242, 2023.

[10] Q. Hu, L. Zhao, J. Wu, K. Gao, D. Luo, Y. Jiang, Z. Zhang, C. Zhu, E. Schaible, A. Hexemer, C. Wang, Y. Liu, W. Zhang, M. Grätzel, F. Liu, T. P. Russell, R. Zhu, and Q. Gong, "In situ dynamic observations of perovskite crystallisation and microstructure evolution intermediated from $[PbI_6]^{4-}$ cage nanoparticles," *Nature Communications*, vol. 8, article 15688, 2017.

[11] A. Günzler, M. Ries, E. Mankel, R. Mews, J. Bayer, M. Schwartzkopf, E. M. Herzig, W. Kowalsky, and N. C. Gerhardt, "Shaping Perovskites: In Situ Crystallization Mechanism of Rapid Thermally Annealed, Prepatterned Perovskite Films," *ACS Applied Materials & Interfaces*, vol. 13, no. 13, pp. 15518–15527, 2021.

[12] M. Majewski, S. Qiu, O. Ronsin, L. Lüer, V. M. L. Corre, T. Du, C. J. Brabec, H.-J. Egelhaaf, and J. Harting, "Simulation of perovskite thin layer crystallization with varying evaporation rates," *Materials Horizons*, vol. 12, no. 2, pp. 555–564, 2025.

[13] T. Maschwitz, L. Merten, F. Ünlü, K. O. Brinkmann, and coauthors, "How crystallization additives govern halide perovskite grain growth," *Nature Communications*, vol. 16, article 9894, 2025.

[14] B. Crist and J. M. Schultz, "Polymer spherulites: A critical review," *Progress in Polymer Science*, vol. 56, pp. 1–63, 2016.

[15] L. Gránásy, T. Pusztai, G. Tegze, J. A. Warren, and J. F. Douglas, "On the growth and form of spherulites," *Physical Review E*, vol. 72, article 011605, 2005.
